# Consumption of Endogenous Caspase‐3 Activates Molecular Theranostic Nanoplatform against Inflammation‐Induced Profibrotic Positive Feedback in Pulmonary Fibrosis

**DOI:** 10.1002/advs.202412303

**Published:** 2024-12-17

**Authors:** Qiu‐Ling Li, Xin Chang, Yu‐Mo Han, Zi‐Chao Guo, Yi‐Na Liu, Bin Guo, Chang Liu, Bin‐Rong Yang, Zhong‐Kai Fan, Hu‐Lin Jiang, Xin Chang

**Affiliations:** ^1^ School of Pharmacy Jinzhou Medical University Jinzhou Liaoning 121001 China; ^2^ Liaoning Provincial Key Laboratory of Marine Bioactive Substances Jinzhou Medical University Jinzhou Liaoning 121001 China; ^3^ The First Affiliated Hospital of Jinzhou Medical University Jinzhou Medical University Jinzhou Liaoning 121001 China; ^4^ State Key Laboratory of Natural Medicines China Pharmaceutical University Nanjing Jiangsu 210009 China; ^5^ Jiangsu Key Laboratory of Druggability of Biopharmaceuticals China Pharmaceutical University Nanjing Jiangsu 210009 China; ^6^ NMPA Key Laboratory for Research and Evaluation of Pharmaceutical Preparations and Excipients China Pharmaceutical University Nanjing Jiangsu 210009 China

**Keywords:** caspase‐3, diagnosis synergistic treatment, molecular diagnosis, precise therapy, pulmonary fibrosis

## Abstract

The limited and backward diagnostic approaches elicit high mortality associated with pulmonary fibrosis (PF) because they fail to identify injury phase of PF. Developing a precisely theranostic nanoplatform presents a promising shortcut to reverse PF. Herein, a specific molecular nanotheranostic (Casp‐GNMT), which is triggered by endogenous cysteinyl aspartate specific proteinase‐3 (caspase‐3), boosts antifibrotic efficacy through bioimaging synergistic with chemotherapy at molecular level, facilitating by ionizable lipid and reactive oxygen species sensitive lipid for precise and manageable therapy. The activation of molecular imaging probe (pCY‐pairs) by consumption of endogenous caspase‐3 initiates fluorescence resonance energy transfer‐guided theranostic pattern, aiming to restore mitochondrial dysfunction‐induced oxidative stress and inflammatory responses in alveolar epithelial cells II (AECs II). This process sequentially resists the expression of interleukin‐1β and vascular endothelial growth factor receptor through combined with nintedanib, further suppressing abnormal injury of AECs II and persistent migration and proliferation of inflammatory cells. Especially, the homeostasis of injured AECs II diminishes excessive accumulation of transforming growth factor‐β to restrain myofibroblasts proliferation and collagen deposition, thereby amplifying the possibility of reversing PF. This theranostic nanoplatform is proposed to provide a prompt and exact approach to enhance diagnostic authenticity and treating efficiency through harnessing endogenous indicator for PF reversal.

## Graphical abstract

An innovative molecular theranostic nanoplatform is specifically recognized and activated by endogenous cysteinyl aspartate specific proteinase‐3 in alveolar epithelial cells II, and reverses injury and progressive phases of PF through impeding the occurrence of inflammation‐induced fibrotic positive feedback, guided by deep‐foci bioimaging plus chemotherapy.

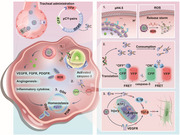

## 1 Introduction

Pulmonary fibrosis (PF), a highly lethal disease with progressive respiratory failure, exhibits alveolar injury and pulmonary parenchyma heterogeneity.^[^ ^1^ ^]^ The behindhand diagnostic methods are known to be the pivotal factors that lead to extremely high mortality rate of PF due to delayed therapy.^[^ ^2^ ^]^ High‐resolution computed tomography (HRCT) is the gold standard of noninvasive diagnostic manner but is limited to identify irreversible advanced phase of PF.^[^ ^3^ ^]^ Notably, the injury phase of PF, which is often reversible and frequently misdiagnosed as pneumonia by HRCT due to overlapping characteristics, resulting in delayed treatment, and accurate identification of this phase of PF typically requires invasive pathological biopsy techniques.^[^ ^4^ ^]^ The advanced phase of PF is more simply recognized, nevertheless, this method is not always compatible with adequately addressed due to irreversible physiological alteration of lung morphology and destruction of pulmonary function resulting from overactivation of pro‐fibrotic pathways and substantial extracellular matrix (ECM) deposition.^[^ ^5^ ^]^ Therefore, there is an urgent need to exploit accurate theranostics that would be able to promptly and efficaciously assess the injury phase of PF, significantly elevating the possibility of PF reversal.

During lung fibrogenesis, sustained injury to alveolar epithelial cells II (AECs II) executes intracellular oxidative stress disproportion, accompanied by biogenetic disorders of mitochondria as well as consequent accumulation of reactive oxygen species (ROS). The elevated concentration of ROS expands the mitochondrial membrane permeability, triggering declined mitochondrial membrane potential, and stimulates cytochrome C release into the cytoplasm, which activates cysteinyl aspartate specific proteinase‐3 (caspase‐3) and induces upregulation of inflammatory cytokines including interleukin‐1β (IL‐1β).^[^ ^6^ ^]^ Thereafter, homeostatic imbalance of AECs II drastically recruits inflammatory cells to infiltrate at the injury site, further motivating overactivation of myofibroblasts and ECM deposition, which exacerbates PF progression.^[^ ^7^ ^]^ During abnormal injury of AECs II, caspase‐3 as an integral substance specifically cleaves aspartate residues of receptor tyrosine kinases (RTKs) to elicit oxidative stress and pro‐inflammatory responses, which is vastly expressed in injured AECs II to launch cellular senescence.^[^ ^8^ ^]^ Consequently, the inhibition of RTKs restricts the substrate availability for caspase‐3 enzyme within the apoptotic pathway, thereby extremely revitalizing the function of injured AECs II and achieving the purpose to reverse PF.

Nintedanib (NIN), a triple tyrosine kinase inhibitor, is generally employed in the clinical medication of PF by hampering epithelial‐mesenchymal transition (EMT) and overactivation of fibroblasts, which can alleviate symptoms in patients, but does not perpetuate survival and reverse PF.^[^ ^9^ ^]^ The limitation arises from oxidative stress imbalance to motivate abnormal mobilization of caspase‐3 in AECs II during injury phase of PF, which handles cell fate and evokes apoptosis and inflammatory responses by cleaving especial substrate, and further incites the overexpression of RTKs in constant injured AECs II.^[^ ^10^ ^]^ Nevertheless, NIN only partially restrains RTKs activity, and fails to efficaciously modulate the senescence and apoptosis of injured AECs II. Given the critical role of homeostasis balance of injured AECs II in PF, we hypothesize that consumption of endogenous caspase‐3 to manage normalization in injured AECs II represents an innovative treating program for PF, which can complement the limitations of NIN by alleviating intracellular oxidative stress and disrupting abnormal sensitization of inflammatory pathways in fibrotic microenvironment.

Accumulating evidence emphasizes that the activated caspase‐3 is regarded as direct biomarkers in injured and senescent cells to develop molecular imaging techniques for monitoring and evaluating the advancement of several cancers.^[^ ^11^ ^]^ The bioprobe is extensively deployed in visual fluorescence imaging for diagnosis of disease through transferring energy from donor fluorophore to acceptor fluorophore, known as fluorescence resonance energy transfer (FRET), exemplified by FRET pair of cyan fluorescent protein (CFP) and yellow fluorescent protein (YFP).^[^ ^12^ ^]^ Therefore, the design of molecular diagnostic probe in accordance with consuming caspase‐3 as activable targets is definitive procedure for the exact diagnosis and treatment of injury phase of PF.

**Figure 1:**
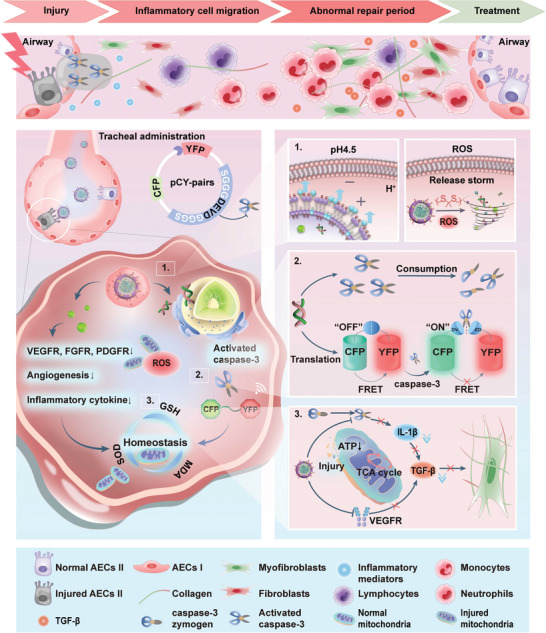
Schematic illustration of therapeutic mechanism for PF reversal. Caspase‐3 activable molecular theranostic nanoplatform (Casp‐GNMT) with dual responsive of ROS/pH realizes PF reversal based on precise diagnosis synergistic with treatment.

In this study, we explore the role of caspase‐3 associated with inflammation and the impact on the progression of fibrotic responses, and the theranostic nanoplatform (Casp‐GNMT) with dual responsive characters of ROS/pH is developed, activated by consumption of caspase‐3 on the basis of previous valuable transfected liposomes for initiating accurate and real‐time recognition as well as monitoring and conclusive therapy in injury and progressive phases of PF.^[^ ^13^ ^]^ The dual responsive profiles of Casp‐GNMT stem from the composition of ionizable lipid (1,2‐dioleoyl‐3‐trimethylammonium‐propane chloride, DOTAP) and ROS‐sensitive thioketal‐linked helper phospholipid [1, 2‐distearoyl‐sn‐glycero‐3‐phosphoethanolamine‐thioketal‐poly(ethylene glycol)_2000_, DSPE‐TK‐PEG_2000_], which payloads CFP (donor)‐YFP (acceptor) plasmid comprised caspase‐3 precisely responsive cleavable peptide of Ser‐Gly‐Gly‐Gly‐Asp‐Glu‐Val‐Asp‐Gly‐Gly‐Gly‐Ser (SGGGDEVDGGGS) (pCY‐pairs) and NIN, manifesting thorough capability of lysosomal escape and ordered release of pCY‐pairs and NIN in response to endogenous ROS‐responsive manner, and achieving favorable transgenic efficiency and extended circulation lifetime. The pCY‐pairs are sufficiently expressed as YFP‐SGGGDEVDGGGS‐CFP in cytoplasm, where activated caspase‐3 cleaves the ‐DEVD‐ and generates fluorescence of CFP and YFP through FRET “OFF” meaning of diagnosis “ON”, performing precise diagnosis of injury and progressive phases of PF. Concurrently, Casp‐GNMT recovers injured AECs II normalization by relieving mitochondrial dysfunction induced‐oxidative stress and restraining inflammatory responses activation through consumption of caspase‐3, finally preventing migration and infiltration of inflammatory cells. To further, the utilization of NIN can suppress myofibroblasts proliferation and EMT of AECs II, accompanying with regulation of injured AECs II normalization with pCY‐pairs to realize PF reversal (**Figure** **1**). Briefly, Casp‐GNMT is a precise, immediate, and capable molecular theranostic nanoplatform for recognition of fibrotic foci, which provides a promising approach to resist inflammation‐induced profibrotic feedback, facilitating the reversal of injury and progressive phases of PF through molecular bioimaging of pCY‐pairs synergistic with chemotherapy of NIN, demonstrating potent employments of monitoring, diagnosis, and precise remedy in lung injury.

## 2 Results

### 2.1 Differences in the Expression of Profibrotic Genes Between Normal and PF Model

**Figure 2:**
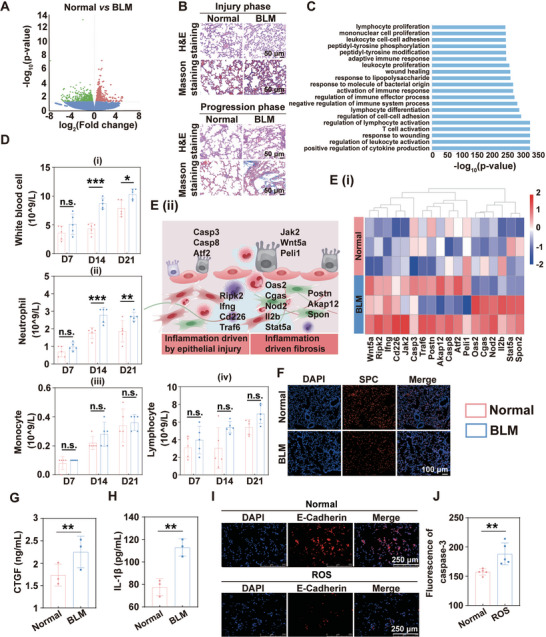
Molecular mechanisms of fibrogenesis in PF mice compared to wide‐type mice. A) Differential gene analysis was performed by volcano plot between wide‐type mice and PF mice (*n* = 3). B) H&E and Masson staining in BLM and Normal groups. C) The enrichment analysis was described by GO database. D) The proliferation of inflammatory cells (*n* = 5), including white blood cell (i), neutrophil (ii), monocyte, (iii) and lymphocyte (iv). E) The differential genes between BLM and Normal by GO analysis and schematic illustration. F) Expression level of SPC by immunofluorescent staining. G,H) Content of CTGF and IL‐1β by ELISA assay (*n* = 3). I) Expression level of E‐Cadherin by immunofluorescent staining. J) Expression level of caspase‐3 by ICW (*n* = 5). The data in each panel was represented as mean ± SD by Student's t test, No significant difference (n.s.): *p* > 0.05, *p < 0.05, **p < 0.005, ***p < 0.001.

There is an ambiguous definition of the pathogenesis of PF, the prolonged injury of AECs II is considered to be the fundamental pathological mechanism, resulting in reduced regeneration ability of lung tissue. Moreover, long‐term injured conditions facilitate the incident of EMT, overactivation of myofibroblasts, and ECM deposition. Eventually, irreversible PF is formed.^[^ ^14^ ^]^ To discover the pivotal factors that extended the development of PF at molecular level, RNA sequencing based on transcriptomics strategies was explored on normal mice and bleomycin sulfate (BLM) induced injury phase of mice. The results evidenced that a huge number of different genes had notable changes, competed with the normal group, the expression levels of 563 genes were upregulated and 576 genes were downregulated in BLM group (**Figure** **2**A). According to the results of H&E staining, there was extensive infiltration of inflammatory cells in BLM group of injury phase compared to progressive phase, demonstrating that the proliferation and migration of inflammatory cells were intensive in injury phase to accelerate PF progression. And ECM deposition increased with PF progression (Figure 2B). Based on the results of the differential genes and histopathology, Go Ontology (GO analysis) was manipulated to perform enrichment analysis, indicating that the progression of PF was closely relevant to inflammatory responses, including proliferation and activation of immune cells and interaction, especially lymphocytes, mononuclear cell and leukocyte cell‐cell adhesion and binding of cytokines (Figure 2C). The results confirmed the hypothesis that aberrant inflammatory responses were the biocatalyst to strengthen the advancement of PF, which exceptionally involved in the formation of fibrosis by promoting immune cells activation of innate and adaptive immune responses and cytokine expression levels.^[^ ^15^ ^]^ We further investigated cells migration and proliferation associated with inflammatory responses by analyzing whole blood of C57BL/6J mice. The results presented that the proliferation of white blood cell, neutrophil, monocyte and lymphocyte in the BLM group showed a time‐dependent increase, implying that inflammation drove the occurrence and development of PF, while the activation of lymphocyte further demonstrated that inflammatory response ran through the whole process of PF development (Figure 2D). To further, we explored the expression levels of differential genes by GO analysis between BLM and Normal, and the results showed that, except for the abnormal expression of genes related to cell migration and invasion (Wnt5a, Postn, and Spon2), and apoptosis (Ripk2, Casp3, and Atf2), the expression level related to immune response in BLM group was significantly higher than that in Normal group (Figure 2E). Surfactant protein C (SPC), the essential proliferative marker of AECs II, was decreased in BLM group than Normal, demonstrating that inhibition of AECs II proliferation was a pathological mechanism in the PF progression (Figure 2F). And the content of connective tissue growth factor (CTGF) and IL‐1β was obviously raised in BLM group, which was always released by injured AECs II to accelerate activation of inflammatory and fibrotic responses, and was assessed by enzyme‐linked immunosorbent assay (ELISA assay) (Figure 2G,H). In addition, the expression of caspase‐3 and epithelial Cadherin (E‐Cadherin) was increased in injured AECs II by detection of immunofluorescent staining and in‐cell western (ICW) (Figure 2I,J). The results illustrated that injured AECs II might promote excessive activation of inflammatory response through the secretion of caspase‐3 and other cytokines, and participated in the occurrence and progression of PF. Therefore, regulating the functional normalization of injured AECs II and further blocking the activation of inflammatory response might be of great significance for the reversal of PF.

### 2.2 Construction and Characterization of ROS/pH‐Responsive Theranostic Nanoplatform

**Figure 3:**
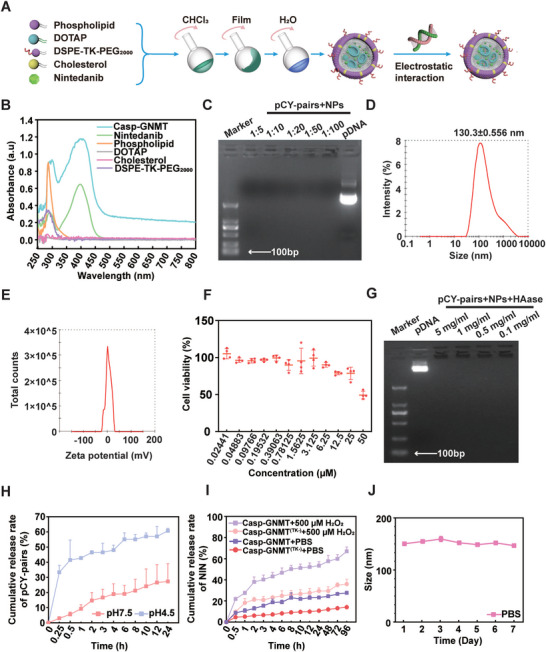
Construction and characterization of Casp‐GNMT. A) The schematic of the preparation of Casp‐GNMT. B) UV–vis spectra of Casp‐GNMT and component excipients at 200–800 nm. C) The loading efficiency of pCY‐pairs on Casp‐GMT using agarose gel electrophoresis. D) Particle sizes of Casp‐GNMT. E) Zeta potential of Casp‐GNMT. F) The biocompatibility of Casp‐GNMT is denoted by cell viability (*n* = 4). G) The sensitivity of Casp‐GNMT to HAase by agarose gel electrophoresis. H) Cumulative release rate of pCY‐pairs in Casp‐GNMT in conditions of pH 4.5 and pH 7.5 (*n* = 3). I) Cumulative release rate of NIN in Casp‐GNMT and Casp‐GNMT^(TK‐)^ in conditions of PBS and 500 µm H_2_O_2_ (*n* = 3). J) The stability of Casp‐GNMT in PBS solution within 7 days (*n* = 3).

The caspase‐3 activatable nanotheranostics loading NIN and molecular imaging probe (pCY‐pairs) were prepared by thin‐layer dispersion method, NIN and pCY‐pairs were payload in Casp‐GNMT (**Figure** **3**A). The ultraviolet–visible (UV–vis) spectra displayed that the characteristic peak of NIN in Casp‐GNMT was indiscriminate with free NIN, which indicated that NIN was successfully loaded into the preparations (Figure 3B). The result of agarose gel electrophoresis proved that pCY‐pairs could be completely loaded into Casp‐GNMT at different ratios (Figure 3C). The particle sizes of Casp‐GNMT were 130.3 ± 0.556 nm by Zetasizer Nano and showed uniform and spherical‐like by transmission electron microscopy image (TEM) (Figure 3D; Figure S1A, Supporting Information). The zeta potential of Casp‐GNMT was near‐neutral after loading electronegative plasmid of pCY‐pairs (Figure 3E). Moreover, DOTAP was a cationic lipid with a risk of cytotoxicity, and the result of cell viability evinced that there was non‐toxic within the range of concentrations of 5 µm used in AECs II (Figure 3F). To analyze the ability to function over the long term, we further explored the sensitivity of Casp‐GNMT to hyaluronidase (HAase), a protease popularly presented in the body. The result represented that Casp‐GNMT could adequately protect pCY‐pairs in the presence of HAase of different concentrations (Figure 3G). DOTAP in Casp‐GNMT was a permanent cationic lipid and contained a quaternary ammonium group, which was electrically neutral at pH 7.0, while positively charged at pH 4.5, indicating that DOTAP held the properties of an ionizable lipid, which was helpful to Casp‐GNMT carrying pCY‐pairs and NIN to escape from the lysosomes (Figure S1B,C, Supporting Information). As clarified in Figure 3H, the result implied that Casp‐GNMT exhibited capable release of pCY‐pairs and NIN at pH 4.5. Alternatively, thioketal (TK)‐linked DSPE‐TK‐PEG_2000_ triggered the release of therapeutics by Casp‐GNMT in response to high ROS environments of 500 µm compared to Casp‐GNMT^(TK‐)^ (Figure 3I). The dual responsive characteristics of ROS/pH motivated the productive release of pCY‐pairs and NIN, which was the premise of achieving the coordination of precise diagnosis and treatment. After that, the particle size changes of Casp‐GNMT were detected to assess the range of application in the physiological environment, illuminating excellent stability in PBS within 7 days (Figure 3J).

### 2.3 Casp‐GMT Theranostic Enables Efficient Delivery and Precise Diagnosis

**Figure 4:**
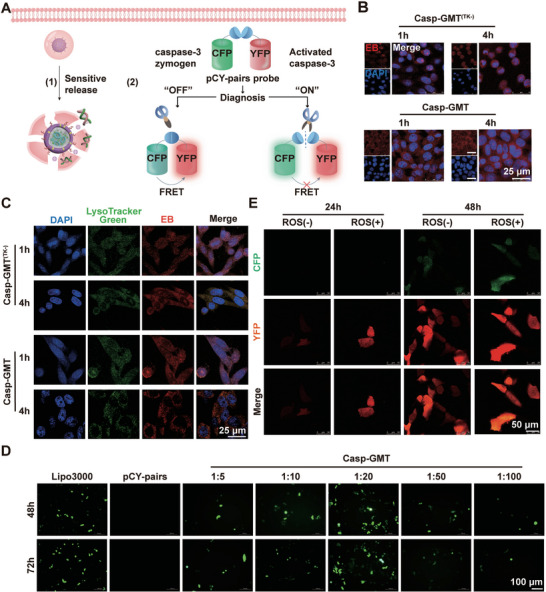
The diagnostic mechanism of Casp‐GMT. A) Diagnostic illustration of Casp‐GMT. B,C) The uptake efficiency and lysosome escape capacity of Casp‐GMT and Casp‐GMT^(TK‐)^ at 1 and 4 h by CLSM. D) The selection of the optimal transfection efficiency of Casp‐GMT. E) Diagnostic sensitivity of pCY‐pairs in the models of wild type and oxidative stress in vitro.

The schematic of **Figure** **4**A illustrates influential position of Casp‐GMT theranostics in precise diagnosis and treatment in injured AECs II. The successful uptake and lysosomal escape are the prerequisites for genes and drugs to exert their effects. Here, we observed uptake efficiency of Casp‐GMT (loading pCY‐pairs only) and Casp‐GMT^(TK‐)^ (DSPE‐mPEG_2000_ contained and loading pCY‐pairs only as control group) by confocal laser scanning microscope (CLSM), which could be actively uptake in a time‐dependent manner (Figure 4B; Figure S2A, Supporting Information). Additionally, Casp‐GMT could quickly escape from lysosome of injured AECs II within 4 h (Figure 4C; Figure S2B, Supporting Information). The lysosomal escape efficiency of Casp‐GMT was higher than that of Casp‐GMT^TK(‐)^, which was beneficial for therapeutic efficacy of diagnosis in combination with treatment in vivo. The pCY‐pairs was a genetically engineered plasmid that linked CFP and YFP via the sensitive sequence of caspase‐3, and productive transfection of pCY‐pairs into injured AECs II was the first step toward accurate diagnosis. The results presented that pCY‐pairs were extremely expressed based on previous preparation at the ratio of 1:20, which was exploited for the following study (Figure 4D). Next, the precise diagnosis of pCY‐pairs with the aid of Casp‐GMT was observed by CLSM in injured AECs II. We discovered that pseudo‐yellow color had appeared, which was superimposed by pseudo‐green of CFP and pseudo‐red of YFP in injured AECs II and only the fluorescence of YFP could be caught in normal AECs II. The reason was that sensitive linker of ‐DEVD‐ could be cleaved from caspase‐3 overexpression in injured AECs II induced by 500 µm H_2_O_2_ and both fluorescence intensities of YFP and CFP were identified at 48 h. Nevertheless, lack of caspase‐3 expression in normal cells resulted in DEVD between CFP and YFP in pCY‐pairs not being blocked, inducing energy transfer from CFP to YFP, which showed YFP fluorescence based on FRET phenomenon (Figure 4E). Alternatively, the content of caspase‐3 was exhibited inhibitory trend in TK(+) group, implying that precision diagnosis was achieved by consuming caspase‐3 (Figure S3, Supporting Information). The results of in vitro studies fully proved that Casp‐GMT could quickly and authentically diagnose caspase‐3 induced by oxidative stress in AECs II, which was requisite for reversing the injury phase of PF.

### 2.4 Regulation of Oxidative Stress Reduces AECs II Injury and PF Progression

**Figure 5:**
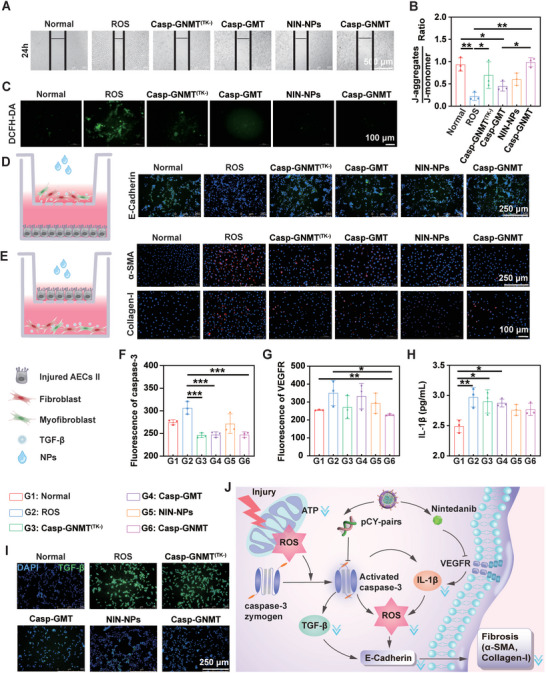
Antifibrosis effect of Casp‐GNMT on diagnosis synergistic with treatment. A) Inhibition of the migration of injured AECs II after treating with various preparations for 48 h. B) Changes of mitochondrial membrane potential by detection kit (*n* = 3). C) ROS content in different treating groups by inverted fluorescent microscope. D) Schematic of coculture model of myofibroblasts in the upper chamber and injured AECs II in the lower chamber (Left). The expression of E‐Cadherin in injured AECs II after treatments by immunofluorescent staining (right). E) Schematic of coculture model of injured AECs II in the upper chamber and myofibroblasts in the lower chamber (Left). The expression of α‐SMA and collagen‐I in myofibroblasts after treating with various formulations (Right). F–H) Fluorescence quantitative analysis of caspase‐3 (F) and VEGFR (G); and IL‐1β (H) by ELISA assay after treating with various formulations (*n* = 3). I) The expression of TGF‐β after incubating with Casp‐GNMT and others in injured AECs II by immunofluorescent staining. J) Schematic of therapeutic mechanism of Casp‐GNMT. The data was represented as mean ± SD by one‐way ANOVA. *p < 0.05, **p < 0.005, ***p < 0.001.

The abnormal injury of AECs II produces irregular profibrotic factors to motivate myofibroblasts overactivation and mechanically accelerates the initiation and formation of PF, which is indicative of the importance to regulate the normalization of injured AECs II in reversing PF. To verify this hypothesis, we first examined whether the administration of Casp‐GNMT could attenuate transformation of profibrotic phenotype of injured AECs II. The treating groups included oxidative stress group (ROS), Normal group, loading pCY‐pairs only (Casp‐GMT), loading NIN only (NIN‐NPs), loading pCY‐pairs and NIN and contained DSPE‐mPEG_2000_ [Casp‐GNMT^(TK‐)^], loading pCY‐pairs and NIN and contained DSPE‐TK‐PEG_2000_ (Casp‐GNMT). The results proved that treating with Casp‐GNMT depressed EMT than control groups by wound healing assays, indicating that Casp‐GNMT had the strongest inhibitory effect for PF progression (**Figure** **5**A). The ability of Casp‐GNMT to mitigate deviant oxidative stress of injured AECs II was investigated by detection of the changes in mitochondrial membrane potential. The results of fluorescence images indicated that Casp‐GNMT possessed strong ability to supervise oxidative stress of injured AECs II at the mitochondrial level. Compared with administration of control treatments, Casp‐GNMT could productively adjust mitochondrial membrane potential of dysfunctional mitochondria and thus hinder injured AECs II migration (Figure 5B). At the cellular level, H_2_O_2_ stimulation could induce the accumulation of ROS to facilitate the occurrence of oxidative stress, and the weaken in mitochondrial membrane potential also suggested excessive ROS accumulated in injured AECs II. The ROS content was further judged using 2′,7′‐dichlorodihydrofluorescein diacetate (DCFH‐DA) to evaluate mitochondrial function after treating with various formulations. The results revealed that ROS in Casp‐GNMT was clearly lessened than that in the controls (Figure 5C). To further explore the mechanism of action of EMT in the progression of PF, the coculture model was developed using transwell device to investigate the interaction mechanism between myofibroblasts overactivation and EMT of injured AECs II. The results signified that the crosstalk between injured AECs II and overactivated myofibroblasts was broken by evaluating the expression of E‐Cadherin, α‐smooth muscle actin (α‐SMA), and collagen‐I, demonstrating that Casp‐GNMT could inhibit EMT and overactivation of myofibroblasts than other treatments (Figure 5D,E). Then, the synergistic effect of pCY‐pairs and NIN was further investigated by repairing injured AECs II homeostasis. Based on preliminary study, EMT was supposed to be associated with oxidative stress in injured AECs II, and caspase‐3, a ROS‐induced proapoptotic protease, was definitely cut down in Casp‐GNMT group rather than Casp‐GMT and NIN‐NPs, indicating that the strategy of diagnosis with treatment could receive precise regulation of injured AECs II normalization, further decreasing myofibroblasts activation and ECM deposition (Figure 5F). Simultaneously, this strong synergistic effect could strengthen the inhibitory effect of NIN on vascular endothelial growth factor receptor (VEGFR), a tyrosine kinase receptor (Figure 5G). In addition, Casp‐GNMT alleviated inflammatory responses than other treating groups in injured AECs II, including IL‐1β at the downstream of caspase‐3 to intercept the progression of PF (Figure 5H). Even more remarkable, the expression of transforming growth factor‐β (TGF‐β), a major regulator of PF, was also directly impaired by Casp‐GNMT, which could suppress the connection between injured AECs II and overactivated myofibroblasts (Figure 5I). The synergistic effect of the diagnosis and treatment was illustrated in Figure 5J.

### 2.5 The Effective Accumulation and Precise Diagnostic Performance

**Figure 6:**
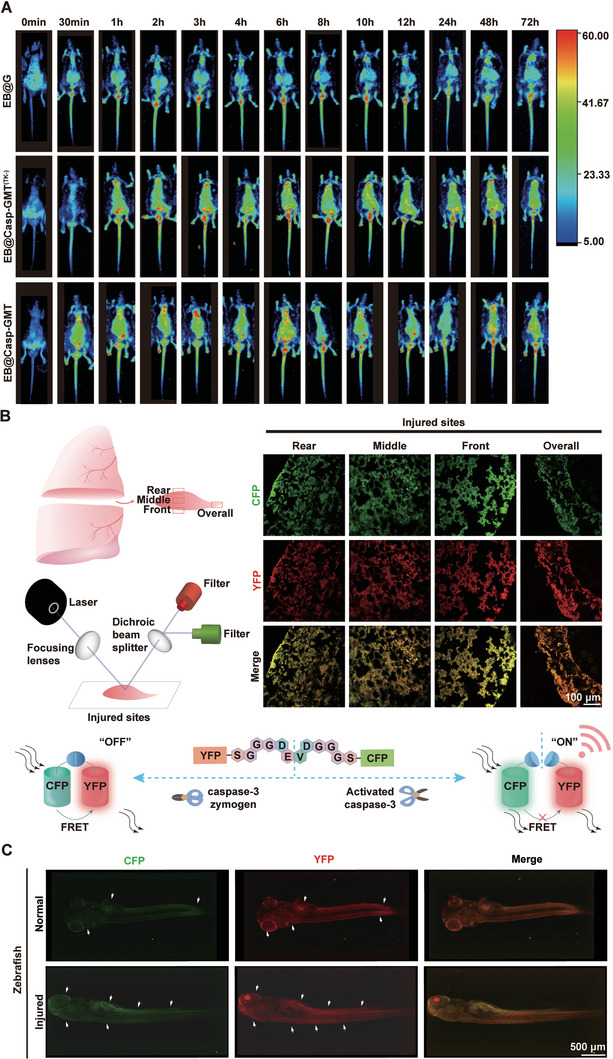
Biodistribution and diagnostic sensitivity of Casp‐GNMT in vivo. A) Biodistribution of EB@G, EB@Casp‐GMT^(TK‐)^, and EB@Casp‐GMT at different time points. B) Diagnostic principle of pCY‐pairs probe (left). Representative images of diagnostic efficiency of Casp‐GMT by two‐photon CLSM in the lung tissues of PF mice (right). C) Diagnostic sensitivity of Casp‐GMT in the models of zebrafish of wild type and oxidative stress.

To determine whether Casp‐GNMT could actively accumulated at lung tissues, we performed in vivo bioimaging experiment with ethidium bromide (EB) labeled free pCY‐pairs (EB@G), EB labeled ROS/pH‐sensitive release nanoplatform loaded with pCY‐pairs (EB@Casp‐GMT) and EB labeled non‐responsive nanoplatform loaded with pCY‐pairs [EB@Casp‐GMT^TK(‐)^]. In these three compositions, EB@Casp‐GMT could deliver pCY‐pairs to the lungs at 30 min, which were first observed at lung tissues within 1 h in [EB@Casp‐GMT^TK(‐)^]. Whereas EB@G was hardly recognized within 72 h, indicating that EB@Casp‐GMT could directly deliver pCY‐pairs in the lungs and accumulated for the longest time, more than 72 h (**Figure** **6**A; Figure S4A, Supporting Information). The accumulation of NIN was investigated and reached similar phenomenon in these groups (Figure S4B, Supporting Information). Besides, the precise diagnostic performance of pCY‐pairs probe loaded into Casp‐GMT was examined to assess FRET phenomenon occurred in injured AECs II of lung tissues by two‐photon CLSM. The results presented that the fluorescence of CFP with pseudo‐green color and YFP with pseudo‐red color, and merged pseudo‐yellow color, were both activated in lung tissues of PF mice, which clarified that overexpressed caspase‐3 in injured AECs II could break amino acid bonding (‐DEVD‐) to restrain energy transfer from CFP chromophores to YFP chromophores, showing a state of diagnostic “ON” based on FRET principle in PF mice (Figure 6B). As shown in Figure S4C (Supporting Information), the expression level of caspase‐3 was significantly reduced before/after treatment of Casp‐GNMT by ELISA assay in injury and progressive phases of PF, demonstrating that Casp‐GNMT could treat PF in relation to consume caspase‐3. Furthermore, the diagnostic sensitivity of Casp‐GNMT in injury and progressive phases of PF was also examined, indicating that the phenomenon of diagnostic “ON” was performed by consuming caspase‐3 in injury and progressive phases of PF (Figure S4D,E, Supporting Information). The expression level of caspase‐3 in injured AECs II was detected by fluorescence‐activated cell sorting (FACS) and immunofluorescent staining, and the results showed that the expression level of caspase‐3 (CASP3 marker) in AECs II (SPC marker) of PF tissue was significantly decreased after treating with Casp‐GMT compared to BLM group, which was similar to Normal, and immunofluorescent staining demonstrated a similar trend (Figure S4F,G, Supporting Information). In addition, to completely exemplify the feasibility in the absence of organ detachment and ameliorate clinical translation possibilities of Casp‐GNMT, the zebrafish was as a model and simulated oxidative stress by n‐butanol. The results demonstrated that other than the Normal group, the n‐butanol stimulated group had pseudo‐yellow fluorescence that formed by overlapping the pseudo‐green of CFP and the pseudo‐red of YFP (Figure 6C). These results bared that caspase‐3 could absolutely identify and cleave sensitive fragments in pCY‐pairs at injured sites, and achieved FRET‐guided accurate diagnosis in injury phase of PF.

### 2.6 Casp‐GNMT Attenuated Migration of Inflammatory Cells and Cytokine Storm and Suppressed Fibrotic Progression in Injury Phase of PF

**Figure 7:**
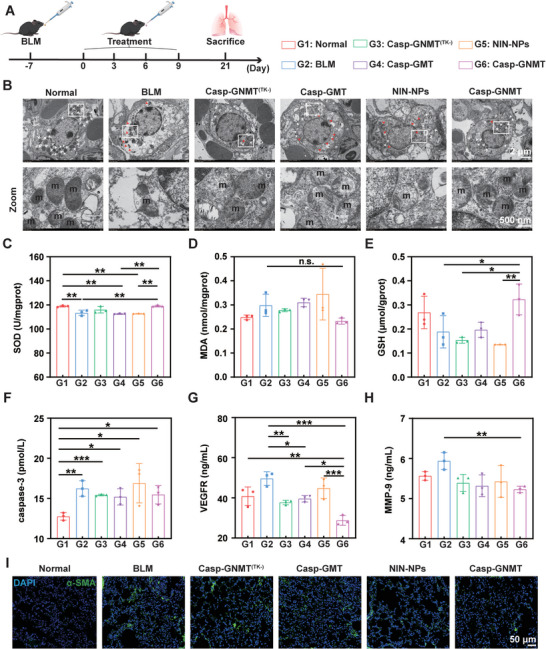
Antifibrosis effect of Casp‐GNMT in injury phase of PF. A) Schematic of PF model construction and treatment in injury phase of PF. B) Morphologies of mitochondria after treating with different groups using TEM. C–E) The content of relative indicators of antioxidant stress by detection kits, SOD (C), MDA (D), GSH (E). F,G) Expression of caspase‐3 and VEGFR by ELISA assay. H) Expression level of MMP‐9 in lung tissues. I) Expression of α‐SMA by immunofluorescent staining. The data was represented as mean ± SD by one‐way ANOVA. No significant difference (n.s.): *p* > 0.05, *p < 0.05, **p < 0.005, ***p < 0.001.

The Casp‐GNMT impeded fibrotic promotion and reversed injury phase of PF through a two‐step procedure: caspase‐3 mediated precise diagnosis by pCY‐pairs probe in the first step followed by unique chemotherapy by theranostic nanoplatform in the second step. To identify the treating efficacy of Casp‐GNMT for PF reversal, we established injury phase of PF mice model by tracheal administration of BLM (**Figure** **7**A). The microstructure of the mitochondria after therapy also supported this conclusion that the ability of Casp‐GNMT could heal injured AECs II. The results illustrated that the quantities of mitochondria of AECs II were markedly shortened in BLM group matched Normal group, the morphologies of mitochondrial were irregular and the volume was progressively increased. Meanwhile, the cristae were gradually disordered, and some of the membrane was broken, resulting in the mitochondria appearing with vacuolization. After treating with different formulations, the morphologies of mitochondrial were noticeably restored in Casp‐GNMT group than that in the others, of which the double membranes of mitochondria had back into the normal form (Figure 7B). Moreover, the quality of mitochondria was attentively referred to intracellular oxidative stress, and superoxide dismutase (SOD), malondialdehyde (MDA) and glutathione (GSH) were determined by detection kits, which implied that Casp‐GNMT had a strong effect to relieve oxidative stress of injured AECs II by up‐regulation of the content of SOD and GSH, and down‐regulation of MDA (Figure 7C–E). The caspase‐3, a vital downstream effector of oxidative stress and upstream master of inflammatory responses, was seriously attenuated in diagnosis synergistic with treatment group, demonstrating that Casp‐GNMT illustrated the best capabilities to suppress injured AECs II apoptosis and systematize cellular homeostasis competed with other treatments and BLM group (Figure 7F).

Besides, oxidative stress inequality would proliferate EMT‐mediated migration by overexpression of VEGFR in injured AECs II. To estimate inducements to EMT, the content of VEGFR was examined by ELISA assay. The results signified that Casp‐GNMT could operatively impede the production of profibrotic factors (Figure 7G). In the progression of PF, the extreme enrichment of ECM was the major factor to expedite the formation of PF. The matrix metalloproteinase‐2 (MMP‐2) and MMP‐9 were assessed to estimate collagen deposition in the PF mice tissues. The results appeared that MMP‐2 and MMP‐9 were apparently diluted in Casp‐GNMT than other preparations, indicating of which could helpfully downregulate ECM accumulation to block PF progression (Figure 7H; Figure S5, Supporting Information). Moreover, the expression level of profibrotic marker of α‐SMA was performed to assess the proliferation of myofibroblasts by immunofluorescent staining analysis. It was exposed that the expression of α‐SMA in Casp‐GNMT group was distinctly lower than that in the BLM group and other treating groups, which was close to the Normal group (Figure 7I). These phenomena strongly supported our hypothesis that regulation of injured AECs II could treat PF through attenuating inflammatory responses and further inhibiting myofibroblasts activation and ECM deposition. According to these favorable results, Casp‐GNMT exposed considerable anti‐PF potential and application prospect based on the pattern of diagnosis in combination with treatment.

### 2.7 Mechanisms of Action on Diagnosis Synergetic Treatment Examined by RNA‐Seq Analysis

**Figure 8:**
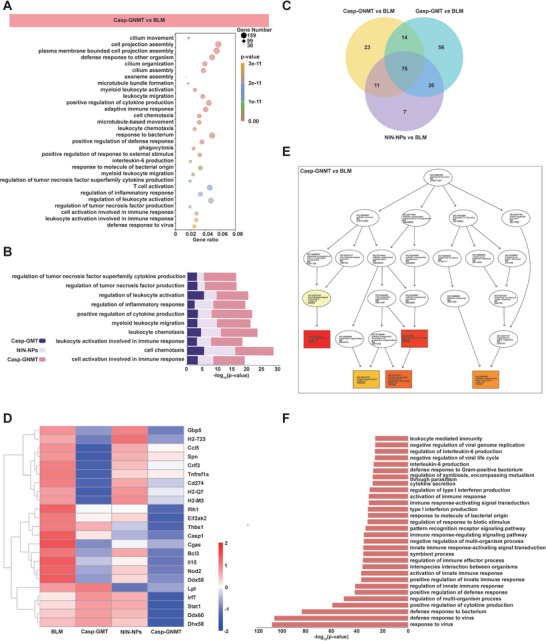
The therapeutic mechanism of Casp‐GNMT in precise theranostic manner. A) The top 30 terms in BP between Casp‐GNMT group and BLM group. B) The differential genes proportion of the top 10 terms in BP associated with inflammation based on GO analysis in Casp‐GNMT, Casp‐GMT, and NIN‐NPs. C) Venn diagrams showed the distribution of differential genes in Casp‐GNMT versus BLM, Casp‐GMT versus BLM, and NIN‐NPs versus BLM in the item of positive regulation of cytokine production. D) Gene cluster analysis in the item of positive regulation of cytokine production among Casp‐GNMT, Casp‐GMT, NIN‐NPs, and BLM. E) The action mechanism of antifibrosis in Casp‐GNMT versus BLM. F) The synergistic mechanism of pCY‐pairs and NIN in Casp‐GNMT versus BLM based on enriched items in BP.

To understand the mechanism of reversing PF based on combination of pCY‐pairs with NIN, we performed RNA‐seq analysis to observe the therapeutic mechanism of antifibrosis. As shown in **Figure** **8**A, the total number of differential genes in Casp‐GNMT group versus BLM were 2780, of which 17 of the top 30 in BP were directly related to inflammation, demonstrating that therapeutic mechanism of Casp‐GNMT was impressively influenced in inflammatory responses. The term in BP associated with immune response expressed distinct mode of action in Casp‐GNMT versus BLM and various formulations versus BLM, demonstrating that reversal mechanism for PF of Casp‐GNMT was different from other treatments (Figure S6, Supporting Information). The enrichment of differential genes of the inflammatory responses was presented in Casp‐GNMT versus BLM, Casp‐GMT versus BLM and NIN‐NPs versus BLM, and the results manifested that plenty of terms in BP that altered were most enriched in the production of cytokines and lymphocytes activation in the Casp‐GNMT versus BLM but less in the Casp‐GMT versus BLM and NIN‐NPs versus BLM (Figure 8B). In addition, the distribution of differential genes of Casp‐GNMT versus BLM, Casp‐GMT versus BLM, NIN‐NPs versus BLM was reanalyzed using Venn diagrams or gene cluster to evaluate positive regulation of cytokine production pathways and top 10 terms associated with inflammatory responses in BP (Figure 8C; Figure S7A,B, Supporting Information). The results indicated that Casp‐GNMT group had 23 distinct differential genes associated with inflammatory responses of action from Casp‐GMT and NIN‐NPs. And the gene cluster analysis exhibited that the differential genes were predominantly associated with inflammatory responses (Figure 8D). These results revealed that the therapeutic scheme of regulation of inflammatory response was critical to reverse injury phase of PF. To further explore the mechanism of action of pCY‐pairs synergistic with NIN, differential genes of Casp‐GMT, NIN‐NPs, Casp‐GNMT, and BLM were analyzed and enriched in BP of GO database. The results explained that in addition to regulating cilium movement (red), microtubule‐based movement (light yellow) and cilium organization (dark yellow), Casp‐GNMT held a conspicuously strengthened effect on enhancing body defense against other organisms including plasma membrane bounded cell projection assembly (orange), cell projection assembly (red) and defense response to other organism (brownish yellow), matched Casp‐GMT and NIN‐NPs (Figure 8E). The differential gene analysis confirmed that the interaction between pCY‐pairs and NIN propelled the initiation of the protective mechanism (Figure S7C,D, Supporting Information). As well, we correlated the principal part of BP in the GO database, and the differential genes were caught that the BP with more meaningful alteration was accompanied to immune responses (Figure 8F), which was consistent with the previous perspective.

### 2.8 Casp‐GNMT Possessed the Potential to Reverse PF in the Progressive Phase

**Figure 9:**
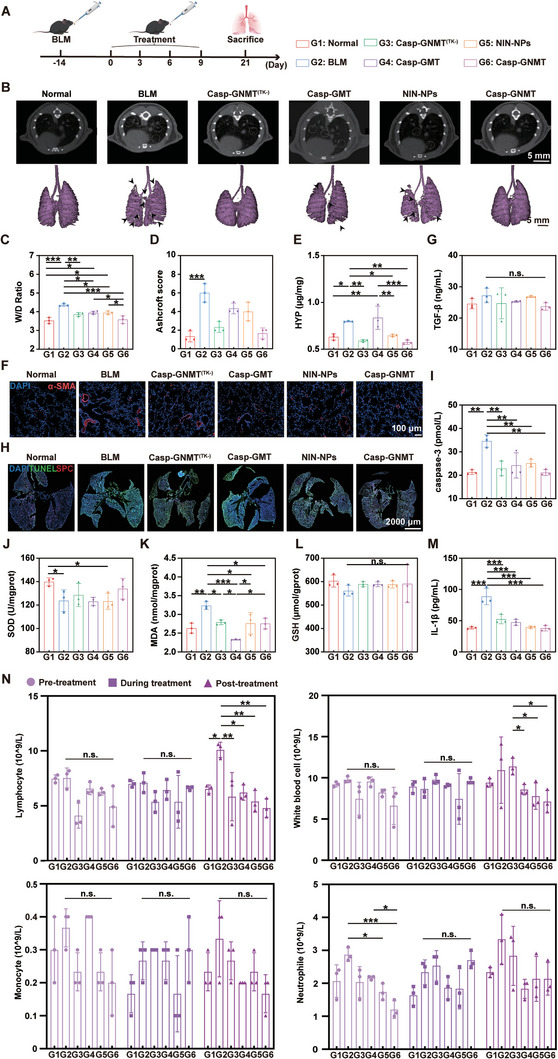
Antifibrosis of Casp‐GNMT in phase of progression of PF. A) Schematic of PF model construction and treatment in progressive phase (*n* = 3). B) Morphologies of PF lungs in vivo by micro‐CT (upper) and 3D reconstruction (lower). C) Evaluation of injury degree by W/D ratio after treating with various formulations. D,E) Therapeutic efficacy in different preparations, Ashcroft score (D), and HYP (E). F) Proliferation of myofibroblasts by immunofluorescent staining. G) Detection of TGF‐β content by ELISA assay. H) Apoptosis of injured AECs II by evaluation of TUNEL level. I) The content of caspase‐3 in lung tissues by ELISA assay. J‐L) Content of oxidative stress‐related indicators, SOD (J), MDA, (K) and GSH (L). M) Content of IL‐1β by ELISA assay. N) The proliferation of recruited inflammatory cells, (lymphocyte, white blood cell, monocyte, and neutrophil) after treating with diverse formulations. The data was represented as mean ± SD by one‐way ANOVA. No significant difference (n.s.): *p* > 0.05, *p < 0.05, **p < 0.005, ***p < 0.001.

This segment was performed to value the theranostic efficacy in progressive phase of PF. The PF mice model was constructed after 14 days of administration of BLM. The treatment lasted 21 days from the completion of the modeling and was administered different preparations for four times (**Figure** **9**A). During treatment phase, the weight of mice was observed to monitor the health conditions of mice. The results displayed that there were no indicative changes in different groups, manifesting good biosecurity of various preparations (Figure S8A, Supporting Information). After treatment of 21 days, micro computed tomography (micro‐CT) imaging analysis was applied to judge the treating efficacy based on the program of diagnosis along with treatment. The white circles represented abnormal shadows in the lungs, lesion sites of PF. And the black arrows displayed areas of the lungs that were not totally ventilated and formed PF. The results declared that Casp‐GNMT had the smallest area of white shadow, indicating the best effect of blocking PF procession than other treating groups. And 3D reconstruction software could more intuitively observe the progression of PF by a ventilation analysis on the PF mice model of various preparations. Combined with the results of CT imaging and lung ventilation analysis, it was turned up that the lung tissue structure had changed and fibrotic lesions had formed in BLM group. In contrast, there was a nearly normal lung structure after treating with Casp‐GNMT, demonstrating that Casp‐GNMT could not only reverse PF in the phase of lung injury, but also treat progressive PF, demonstrating strong ability to reverse PF (Figure 9B). The healthy condition was assessed to discover the injury degree by wet/dry ratio (W/D ratio), showing repairing ability of Casp‐GNMT (Figure 9C). In addition, the result of Ashcroft score was evaluated by analyzing the results of H&E and Masson staining, indicating that Casp‐GNMT had capability to reverse model mice from fibrosis to normal (Figure 9D). And the content of hydroxyproline (HYP) and the expression of α‐SMA could directly reflect the severity of PF, which showed a depressed tendency in Casp‐GNMT than other treating groups, implying that Casp‐GNMT could block PF progression by reducing myofibroblasts overactivation and ECM deposition (Figure 9E,F). The expression level of profibrotic factors, including TGF‐β and VEGFR, was detected by ELISA assay, and revealed efficaciously therapeutic efficacy to suppress the proliferation of myofibroblasts (Figure 9G; Figure S8B, Supporting Information). The inhibition of myofibroblasts moderated ECM deposition and changed in PF lung structure, which could indirectly ease apoptosis of injured AECs II. The expression of SPC, the marker of AECs II proliferation, was determined and represented raised expression level in Casp‐GNMT, which was benefit to treatment of PF (Figure 9H). The apoptotic protease of caspase‐3, was lower after treating with Casp‐GNMT (Figure 9I). And the oxidative stress related factors of SOD and GSH were obviously upregulated and MDA was weakened in Casp‐GNMT group than others, displaying similar lung condition to Normal group (Figure 9J–L). The reinforced antioxidant capacity of injured AECs II diminished the secretion of IL‐1β and further blocked the migration of inflammatory cells in Casp‐GNMT (Figure 9 M,N; Figure S8C, Supporting Information). Furthermore, the biosafety of various treatments was estimated by determining the content of alanine aminotransferase (ALT), aspartate transaminase (AST), creatine kinase (CK), and blood urea nitrogen (BUN), which indicated that the formulations had no toxicity of liver, kidneys and heart tissue (Figure S8D–G, Supporting Information). After treatment of progressive phase of PF, we were able to conclude that Casp‐GNMT could reverse PF at the phases of injury and progression to inhibit the migration of immune cells and activation of inflammatory responses through a synergistic strategy of diagnosis and treatment.

## 3 Discussion

Pulmonary HRCT is universally utilized in the diagnosis of PF, while the application is limited by great differences in pathologic mechanism of different phases of PF.^[^ ^16^ ^]^ Particularly, the injury phase of PF presents a more distinct pathological pathway compared to the advanced phase, offering a viable opportunity for therapeutic interventions aimed at reversing PF. During the injury phase, the dysfunction of mitochondria in AECs II induces oxidative stress and upregulation of caspase‐3, which stimulates the activation of inflammatory responses and the release of pro‐inflammatory cytokines, resulting in injury and even apoptosis of AECs II. This pathological condition can exacerbate myofibroblasts overactivation and abnormal ECM deposition, further facilitating development and formation of PF.^[^ ^17^ ^]^ Therefore, we speculate that regulating homeostasis of AECs II could represent a promising therapeutic approach for injury and progressive phases of PF reversal.

Initially, differential gene expression analysis contrasting injury phase of PF with normal mice establishes perceptibly increased expression of genes associated with inflammatory responses, encompassing innate and adaptive immune responses. Among them, the proportion of genes related to innate immune response is ≈40%, primarily involving the proliferation of immune cells and the secretion of proinflammatory cytokines. According to current literature reports, these phenomena regularly appear in injury phase of PF, and the secretion of pro‐inflammatory cytokines is closely related to intracellular oxidative stress caused by perpetual injury of AECs II. Nonetheless, timely treatment is often hindered by inadequate diagnostic techniques in injury phase of PF, resulting in the development of fibrosis. This clinical challenge has motivated us to explore a novel theranostic policy aimed at correcting pathological condition of overactivation of inflammatory responses by precise diagnosis‐guided treatment. A molecular imaging probe can be employed to explicitly identify pathological microenvironment changes at the molecular level, and which can also estimate abnormal gene or protein levels before morphological changes occur in affected tissues.

In the context of PF in mice, the overexpressed caspase‐3 protease emerges as a compelling target, serving as a nexus between oxidative stress regulation and the inflammatory response in injured AECs II, and functioning as a principal protease in mediating apoptosis.^[^ ^18^ ^]^ Furthermore, we and others have certified a cleavable motif of ‐DEVD‐ motif that is selectively targeted by caspase‐3, and can be peculiarly utilized by directing degradation of ‐DEVD‐ motifs in injured AECs II for a trigger of precise theranostic platform.^[^ ^19^ ^]^ The pathologically sensitive nanoplatform has been developed with high precise theranostic nanoplatform for injury and progressive phases of PF on a molecular level, we draw from insights to fluorescent protein FRET pairs of DEVD motifs conjugated, which are genetically engineered plasmid and can be exactly recognized by caspase‐3 proteases following transfected into fluorescent protein FRET pairs, paving the way for precise diagnosis‐guided treatment. Therefore, we focus on reversing injury and progressive phases of PF by developing a dual responsive of ROS/pH theranostic nanoplatform with caspase‐3 consumption in injured AECs II to play an antifibrotic effect, guided by FRET‐based theranostics.

The rational design of the theranostic nanoplatforms can reverse the injury and progressive phases of PF, which realizes accurate recognition and diagnosis facilitated by caspase‐3‐dependent molecular probe and dominates all cells homeostasis by attenuating inflammatory responses and remodeling fibrotic microenvironment, respectively. Our present work further demonstrates that the nanoplatform can realize precise diagnosis in vitro and in vivo, as shown in different models, including injured AECs II, oxidative stress in zebrafish, and PF mice model at phases of injury and progression of PF. The results reveal that the pCY‐pairs in the nanoplatform are quickly identified and cleaved by the overactivated caspase‐3 after sufficient transfection into YFP‐DEVD motifs‐CFP, resulting in diagnosis “ON” and the separation of CFP and YFP, thus enabling precise and real‐time diagnosis during injury and progressive phases of PF. Subsequently, Casp‐GNMT modulates the homeostasis of injured AECs II, further preventing the recruitment of inflammatory cells, and curbing the overactivation of myofibroblasts and the deposition of ECM through the procedure of pCY‐pairs synergistic with NIN, and finally achieving PF reversal therapy. In addition, Casp‐GNMT exhibits favorable biosafety profiles, characterized by minimal hepatotoxicity, nephrotoxicity, and cardiotoxicity, suggesting substantial potential of Casp‐GNMT for clinical applications, marking an initial step toward clinical implementation. Despite these encouraging results, future work will focus on clinical translation of Casp‐GNMT, which is necessary to refine the restrictions of this study in the clinical application.

## 4 Conclusion

In this study, we propose an innovative strategy for suppression of pulmonary inflammation and resolution of fibrotic foci in PF therapy through the integration of precise diagnostics with chemotherapy. According to our data, Casp‐GNMT highlights the advantages of diagnosis synergistic with treatment to resist PF on inhibition of inflammatory responses, myofibroblasts activation and ECM deposition. Notably, Casp‐GNMT can effectively reverse PF according to efficient transfected performance, diagnostic sensitivity and diagnosis in combination with treatment.

## 5 Experimental Section

### Materials

Lecithin, cholesterol, NIN, Coumarin 6, and BLM were purchased from Shanghai Aladdin Biochemical Technology Co., Ltd. (Shanghai, China). DOTAP and DSPE‐mPEG_2000_ were acquired from A.V.T Pharmaceutical Technology Co., Ltd. (Shanghai, China). DSPE‐TK‐PEG_2000_ was purchased from Xi'an ruixi Biological Tcehnology Co., Ltd. (Xi'an, China). 1,1′‐dioctadecyl‐3,3,3′,3′‐tetramethylindocarbocyanine perchlorate (DiI) was purchased from Xi'an Biolite Biotech Co., Ltd. (Xi'an, China). 4′,6‐diamidino‐2‐phenylindole (DAPI), Roswell park memorial institute 1640 medium (RPMI‐1640), dulbecco's modified eagle medium (DMEM), and DNA Ladder were purchased from Nanjing Keygen BioTECH Co., Ltd. (Nanjing, China). Methylthiazolyldiphenyl‐tetrazolium bromide (MTT) and agarose were purchased from Biofroxx (Einhausen, Germany). Rabbit anti‐SPC, rabbit anti‐TGF‐β, rabbit anti‐E‐Cadherin, rabbit anti‐Collagen‐I, rabbit anti‐α‐SMA, rabbit anti‐caspase‐3, rabbit anti‐VEGFR, Cy3 conjugated goat anti‐rabbit IgG (H + L), FITC conjugated goat anti‐rabbit IgG (H + L) and terminal deoxynucleotidyl transferase‐mediated dUTP‐biotin nick end labeling assay (Tunel assay kit) were purchased from Servicebio Technology Co., Ltd. (Wuhan, China). Lyso‐Tracker Green DND‐26 and Lyso‐Tracker Red DND‐99 were purchased from Yeasen Biotechnology Co., Ltd. (Shanghai, China). HAase was purchased from Shanghai Yuanye Bio‐technology Co., Ltd. (Shanghai, China). EB was purchased from Beijing leagene biotech. Co., Ltd. (Beijing, China). 4% paraformaldehyde was purchased from Biosharp Biotech. Co., Ltd. (Hefei, China). Mitochondrial membrane potential assay kit with JC‐1 was purchased from Beyotime Biotech. Co., Ltd. (Shanghai, China). DCFH‐DA was purchased from Sigma Aldrich (St. Louis, USA). Fetal bovine serum (FBS) was purchased from Gibco (Grand Island, USA). Goat anti‐rabbit IRDye 680 was purchased from LI‐COR Biosciences (Lincoln, Nebraska, USA). SOD assay kit (WST‐1 method), GSH assay kit, MDA assay kit (TBA method), inducible nitric oxide synthase (iNOS), and HYP were purchased from Nanjing Jiancheng Bioengineering Institute (Nanjing, China). Lipofectamine 3000 (lipo 3000) was purchased from Thermo Fisher Scientific (Waltham, Massachusetts, USA). ELISA kits for IL‐1β, TGF‐β, MMP‐2, MMP‐9, VEGFR, caspase‐3, and CTGF were purchased from Nanjing BYabscience biological technology Co., Ltd. (Nanjing, China). The pCY‐pairs probe was purchased from the Public Protein/Plasmid Library (Nanjing, China). The flow antibodies of SFTPC and caspase‐3 were purchased from Beijing Biosynthesis Biotechnology Co., Ltd. (Beijing, China).

### Cell Culture

Human non‐small cell lung cancer cells (A549 cells) and human normal lung epithelial cells (BEAS‐2B cells) were cultured in RPMI‐1640 containing 10% FBS, and NCTC clone 929 (L‐929 cells) were cultured in DMEM medium containing 10% FBS.

### Animals

Male C57BL/6J mice aged 6–8 weeks were purchased from Liaoning Changsheng Biotechnology Co., LTD. (Benxi, China). The feeding conditions were carried out according to the Experimental Animal Center of Jinzhou Medical University, which was placed in a light/dark cycle of 12/12 h. The study involving animals was approved by the Animal Experimentation Ethics Committee of Jinzhou Medical University, and the assigned approval number of the experimental investigator (Qiu‐Ling Li) was 2309019.

### Preparation of Casp‐GNMT, Casp‐GNMT^(TK‐)^, Casp‐GMT, and NIN‐NPs

All the formulations were prepared by thin film dispersion method. Briefly, lecithin, cholesterol, DSPE‐TK‐PEG_2000_, NIN and DOTAP were absolutely dissolved in trichloromethane and removed organic solvents by rotary evaporator. Then, deionized water (10 mL) was added to construct NIN‐NPs. Casp‐GNMT was obtained after adding pCY‐pairs based on NIN‐NPs. The composition of Casp‐GMT was lecithin, cholesterol, DSPE‐TK‐PEG_2000,_ and DOTAP, and other parts were the same as Casp‐GNMT. Casp‐GNMT^(TK‐)^ (control group) was replaced with DSPE‐TK‐PEG_2000_ by DSPE‐mPEG_2000_.

### Loading Efficiency of Casp‐GNMT to pCY‐Pairs

NIN‐NPs and pCY‐pairs were mixed at different mass ratio of 1:5, 1:10, 1:20, 1:50, 1:100 to screen the optimal construction of Casp‐GNMT. Then, the gel electrophoresis experiment was carried out with 10% agarose to verify the loading efficiency of Casp‐GNMT, and electrophoretic conditions for 90 V, 40 min by electrophoresis apparatus (Tanon, HE‐120, China).

### Characterization of Casp‐GNMT

The UV spectra of Casp‐GNMT, lecithin cholesterol, NIN, DSPE‐TK‐PEG_2000,_ and DOTAP were measured by UV–vis spectrophotometer in the wavelength range from 250 to 800 nm. The particle size and zeta potential were measured via Zetasizer Nano (Malvern, Zetasizer Nano ZSE, UK). The morphology of Casp‐GNMT was recorded by TEM (Thermo Fisher Scientific, FEI Tecnai G^2^ F30, USA).

### Stability of Casp‐GNMT in Different Conditions—HAase Condition

Casp‐GNMT (pCY‐pairs: 2 µg) was mixed with different concentrations of HAase and incubated for 1 h to evaluate the stability of pCY‐pairs in HAase environment. The loading efficiency of Casp‐GNMT was performed by gel electrophoresis experiment with 10% agarose, and the free pCY‐pairs was used for blank control. After that, the image of agarose gel was photographed by gel imager (Tanon 2500R, China).

### Stability of Casp‐GNMT in Different Conditions*—*Dispersion Stability

In this experiment, 100 µL of Casp‐GNMT was taken and diluted to 1 mL using PBS, and then the particle size of Casp‐GNMT was measured by Zetasizer Nano within 7 days to appraise the stability of Casp‐GNMT.

### Cumulative Release of pCY‐Pairs and NIN Based on ROS/pH Sensitivity—pCY‐Pairs

Casp‐GMT (pCY‐pairs: 35 µg) was placed into dialysis tube (MWCO: 500), and the releasing mediums were phosphate buffer of pH 4.5 and pH 7.5, respectively. The cumulative release medium was put at 37 °C and was taken out 50 µL at the times of 0.25, 0.5, 1, 2, 3, 4, 6, 8, 10, 12, and 24 h, and then added the new medium of 50 µL. The release medium contained pCY‐pairs was measured by NanoDrop UV–Visible Spectrophotometer (Thermo Fisher, OneC, USA). The cumulative release of pCY‐pairs was calculated according to Equation (1).

(1) $$\begin{array}{ccc} & & T_{n}\left(\%\right)\\ & & =\left\{\left[\left(C_{n}{V_{\mathrm{Release}}}_{\;\mathrm{medium}}+\left(C_{n-1}+C_{n-2}+\cdots \cdots +C_{2}+C_{1}\right)V_{\mathrm{Removed}}\right)\right]/m_{\mathrm{total}}\right\}\\ & & \times 100\% \end{array}$$

### Cumulative Release of pCY‐Pairs and NIN Based on ROS/pH Sensitivity—NIN

NIN‐NPs and NIN‐NPs^(TK‐)^ (200 µL) were placed into dialysis tube (MWCO: 500), respectively, and 3 mL of 500 µm H_2_O_2_ solution was added as the release medium, and PBS solution was as the control group. The release medium of 200 µL was taken out at the times of 0.5, 1, 2, 3, 4, 6, 8, 10, 12, 24, 48, 72, and 96 h. The three parallel groups were collected at different time points. The concentration of the NIN in the release medium was measured at 391 nm using microplate reader (Biotek, Synergy H1 Hybrid, USA). The cumulative release of NIN was calculated according to Equation (1).

### Cell Viability of Casp‐GNMT

A549 cells with the density of 1 × 10^4^ cells per well were seeded into 96‐well plate and cultured overnight at 5% CO_2_ and 37 °C. The Casp‐GNMT with different concentrations was added into the plates and incubated with A549 cells for 48 h. After that, 20 µL of MTT was added and incubated with the cells for 4 h. Then, culture‐medium was carefully removed and 200 µL of DMSO solution was added into each well. After that, the absorbance was determined at 490 nm and cell viability was calculated using Equation (2).

(2) $$\mathrm{Cell}\;\mathrm{viability}\;\left(\%\right)=\left(A_{\mathrm{test}}-A_{\mathrm{blank}}\right)/\left(A_{\mathrm{control}}-A_{\mathrm{blank}}\right)\times 100\%$$

### Uptake Efficiency of Preparations

EB‐labeled Casp‐GMT and EB‐labeled Casp‐GMT^(TK‐)^: A549 cells were cultured with 3 × 10^4^ cells per well in glass bottom dish overnight. Casp‐GMT was prepared according to the described method, and pCY‐pairs were labeled with EB. Casp‐GMT^(TK‐)^ was used as control group. EB‐labeled Casp‐GMT and EB‐labeled Casp‐GMT^(TK‐)^ were incubated with A549 cells for 1 and 4 h, and then removed culture medium and washed dishes for three times using PBS. After that, the cells were fixed with 4% paraformaldehyde for 15 min and continued to wash dishes for three times. The nuclei were stained with DAPI at 37 °C for 15 min and washed dishes for three times. Finally, 1 mL PBS was added to the dish to maintain the cell morphology and observed the uptake efficiency of EB‐labeled Casp‐GMT and EB‐labeled Casp‐GMT^(TK‐)^ by CLSM (Leica, SP5II, Germany).

### Uptake Efficiency of Coumarin 6‐NPs and Coumarin 6‐NPs^(TK‐)^

A549 cells were cultured with 3 × 10^4^ cells per well in a glass bottom dish overnight at 37 °C and 5% CO_2_. The preparation of Coumarin 6‐NPs was same as the described method of NIN‐NPs, while replaced the loaded NIN with coumarin 6, Coumarin 6‐NPs^(TK‐)^ were used as control. Coumarin 6‐NPs and Coumarin 6‐NPs^(TK‐)^ were added into dishes and incubated for 1 and 4 h, respectively. Then, the cultural medium was removed and washed dishes for three times. 4% paraformaldehyde was used to fixed the cells and washed another three times with PBS. The nuclei were stained with DAPI and recorded uptake efficiency by CLSM.

### Lysosome Escape Capacity—pCY‐Pairs

A549 cells were seeded into glass bottom dish with 3 × 10^4^ cells per well and cultured overnight at 37 °C and 5% CO_2_. Casp‐GMT was prepared by method described, and the pCY‐pairs were labeled with EB (EB‐labeled Casp‐GMT). EB‐labeled Casp‐GMT^(TK‐)^ was used as control. Accordingly, EB‐labeled Casp‐GMT and EB‐labeled Casp‐GMT^(TK‐)^ were incubated with cells for 1 and 4 h, respectively. Then, the cells were fixed with 4% paraformaldehyde for 15 min at room temperature and washed the dishes for three times. And the lysosome was labeled by LysoTracker Green DND‐26 (30 µm), and the nucleus was stained with DAPI. After that, the lysosome escaping capacity of pCY‐pairs was observed and recorded using CLSM.

### Lysosome Escape Capacity*—*Drug

Coumarin 6 replaced NIN in this experiment. A549 cells were cultured with 3 × 10^4^ cells per well in a glass bottom dish at 37 °C and 5% CO_2_. Coumarin 6‐NPs^(TK‐)^ was prepared as described method and incubated with A549 cells for 1, 4, and 6 h. And the cells were fixed with 4% paraformaldehyde for 15 min, and then washed A549 cells for three times. The lysosome was stained by Lysotracker Red (200 nm) for 15 min, and the nucleus was labeled with DAPI. Lysosome escape capacity of drug was analyzed using CLSM.

### Gene Transfection Efficiency of Casp‐GMT

A549 cells were seeded into 12‐well plate with 8 × 10^4^ cells per well. Different mass ratio of Casp‐GMT at 1:5, 1:10, 1:20, 1:50, 1:100 between pCY‐pairs and LNPs were added into the plates with 500 µL FBS‐free RPMI‐1640 medium for 4 h, and lipo 3000 was the control. After 4 h, the FBS‐free RPMI‐1640 medium was removed and replaced with RPMI‐1640 medium containing 10% FBS to culture cells for 72 h. The expression of pCY‐pairs was appraised at 48 and 72 h by inverted fluorescence microscopy, respectively (Leica, DMI4000B, Germany).

### Diagnostic Sensitivity of pCY‐Pairs Based on FRET Principle

BEAS‐2B cells were cultured into glass‐bottom dish with 3 × 10^4^ cells per well. The pCY‐pairs: the preparations of 1:20 were shown the best transfection efficiency for following researches. Casp‐GMT was added into cell plate with FBS‐free RPMI‐1640 and incubated for 4 h. And then, the cultural medium was replaced with RPMI‐1640 medium containing 10% FBS for culturing within 48 h in the models of oxidative stress with 500 µm H_2_O_2_ or normal cells. The diagnostic sensitivity of pCY‐pairs was assessed by CLSM at 24 and 48 h. The excitation wavelength of CFP was 458 nm, and the emission wavelength was 460–517 nm. The excitation wavelength of YFP was 514 nm and emission wavelength were >516 nm.

### Wound Healing Assay In Vitro

A549 cells were seeded into six‐well plate with 12 × 10^4^ cells per well and cultured overnight. When the cell density was above 90%, The scratch in cell plates was formed by a 10 µL tip. Oxidative stress with 500 µm H_2_O_2_ and normal cells were positive and negative control, respectively. The cells were treated with Casp‐GNMT, Casp‐GNMT^(TK‐)^, Casp‐GMT and NIN‐NPs for 24 h. The inhibition of cell migration was examined by inverted microscope.

### Detection of Intracellular ROS Content

A549 cells were cultured into six‐well plate with 1 × 10^5^ cells per well for 24 h. Casp‐GNMT, Casp‐GNMT^(TK‐)^, Casp‐GMT, and NIN‐NPs were added into cell plate with 500 µm H_2_O_2_ for 48 h. After that, the cells were incubated with DCFH‐DA probe at 37 °C for 30 min, and the ROS level was detected using inverted fluorescence microscope.

### Immunofluorescent Staining In Vitro

A549 cells were seeded into six‐well plate with 1 × 10^5^ cells per well and cultured overnight at 37 °C and 5% CO_2_. 500 µm H_2_O_2_ was first treated for 24 h to construct oxidative stress model in vitro. Casp‐GNMT, Casp‐GNMT^(TK‐)^, Casp‐GMT, and NIN‐NPs were added into the plate and cultured for 48 h. And then, the cells were fixed with 4% paraformaldehyde for 15 min and washed plate for three times with PBS. After that, the primary antibodies of rabbit anti‐E‐Cadherin (1:1000), rabbit anti‐Collagen‐I (1:1000), rabbit anti‐α‐SMA (1:1000) and rabbit anti‐TGF‐β (1:1000) were incubated at 4 °C overnight and washed plates three times for 5 min each time. And then, the Cy3 or FITC labeled secondary antibody (1:900) was added and incubated with the cells for 4 h. Finally, the protein expression level was recorded with inverted fluorescence microscope.

### Inhibition of EMT by Transwell Assay

L929 cells were placed into the upper chamber of transwell at a density of 2 × 10^4^ cells per well, and A549 cells were placed into the lower chamber with a density of 5 × 10^4^ cells per plate. The cells were pretreated by TGF‐β with the concentration of 50 ng mL^−1^ for 24 h. L929 cells were incubated with different treatments for 48 h. The expression of E‐Cadherin was estimated by immunofluorescent staining, and the primary antibody of rabbit anti‐E‐Cadherin (1:1000) incubated with cells overnight, and then FITC conjugated goat anti‐rabbit IgG (H+L) (1:900) was added and incubated for 4 h. The results were photographed using inverted fluorescence microscopy.

### ICW Assay

A549 cells were seeded into ICW specific 96‐well plate with 1 × 10^4^ cells per well and cultured overnight. The cells were pretreated with 500 µm H_2_O_2_ and then incubated with cells for 48 h. And then, the cells were fixed with 4% paraformaldehyde at room temperature for 15 min and washed three times using PBS. After that, 0.1% Triton X‐100 was used to permeate the cells for 2 h. Then, the primary antibodies of rabbit anti‐caspase‐3 (1:1000) and rabbit anti‐VEGFR (1:1000) were added into the plates and incubated with the fixed cells overnight at 4 °C. And next, the fixed cells were washed with PBS containing 0.1% tween‐20 for four times, and the secondary antibody of goat anti‐rabbit IRDye 680 (1:800) was incubated with the cells for 4 h. Finally, the protein expression level was assessed by Odyssey NIR two‐color fluorescence imaging system (Li‐COR, Odyssey CLX‐0418, USA).

### Biodistribution of Casp‐GNMT, Casp‐GNMT^(TK‐)^, Casp‐GMT and NIN‐NPs

The mice were injected BLM (5 mg kg^−1^) to develop PF mice model by tracheal administration. After that, the PF mice models were administrated by various preparations.

### pCY‐Pairs

EB@Casp‐GMT, EB@Casp‐GMT^(TK‐)^ and EB@G were delivered by tracheal administration (pCY‐pairs: 1.2 mg kg^−1^), and EB‐labeled free genes and EB‐labeled Casp‐GMT^(TK‐)^ were used as controls. The distribution of various treatment in the lungs were observed by multi‐functional small animal in vivo imager (BRUKER, In Vivo FX PRO, USA).

### Drug

DiI was replaced with NIN in biodistribution experiment. DiI labeled Casp‐GNMT, DiI labeled Casp‐GNMT^(TK‐)^ and free DiI were administered (DiI: 1.4 mg kg^−1^). And biodistribution of different formulations was assessed by multi‐functional small animal in vivo imager.

### Transfection Efficiency of pCY‐Pairs In Vivo—Casp‐GMT

The PF mice models were treated with Casp‐GMT by tracheal administration (pCY‐pairs: 1.2 mg kg^−1^). The PF mice were euthanized at different time points, the lungs were removed, and stored at −80 °C. The frozen lung tissues were fixed by 30% sucrose solution, and OTC embedding agent was used for embedding. And then the frozen microtome was used with a section thickness of 10 µm. The lung tissue sections were photographed using a two‐photon CLSM (Olympus Corporation, FV1200, Japan). The emission wavelength of CFP was 458 nm, the emission wavelength of YFP was 527 nm, and the excitation wavelength was 850 nm.

### Possibility of Individualized Diagnosis

The injured models were developed after incubation 12 h of zebrafish embryos using n‐butanol (900 nm). Casp‐GMT was added into the culture to treat injured models of zebrafish embryos and normal zebrafish embryos for 96 h. After that, the fish bodies were fixed by 4% paraformaldehyde for 24 h, and then fixed with 2% agarose and photographed using CLSM. The excitation wavelength of CFP was 488 nm and the emission wavelength was 499–551 nm. The excitation wavelength of YFP was 561 nm, and the emission wavelength was 571–625 nm. Zebrafish embryos was purchased from Eze‐Rinka (China, Nanjing).

### Diagnostic Sensitivity In Vivo by FACS Assay and Immunofluorescent Staining

The mice were euthanized, and the lung tissues were collected, which were placed in a cell filter (70 µm) and was grounded by grinding pestle in PBS solution. After that, the suspension was collected and centrifuged at 4 °C, 800 g for 5 min. The supernatant solution was removed and suspended cells were precipitated with PBS solution to place it in the flow tube. The rabbit anti‐SP‐C/FITC conjugated antibody (2 µL) and rabbit anti‐caspase‐3 p17/PE‐conjugated antibody (2 µL) were added into the tube, respectively, and incubated with the cells at 4 °C for 30 min. After then, the cells were washed using PBS for 3 times, and centrifuged at 4 °C, 800 g for 5 min. The normal lung tissue was prepared as SPC positive tube by incubating with rabbit anti‐SP‐C/FITC, and the BLM induced PF lung tissue was prepared as caspase‐3 positive tube by incubating with rabbit anti‐caspase‐3 p17/PE. The supernatant solution was carefully removed and collected the cells to a new flow tube and tested by FACS machine.

### Treatment of PF Mice—Injury Phase of Lung Tissue

The mice model of injury phase was developed after 7 days of BLM administration. The PF mice model was randomly divided into five groups, including BLM, Casp‐GNMT, Casp‐GNMT^(TK‐)^, Casp‐GMT, and NIN‐NPs, Normal mice were as control group (*n* = 5). The PF mice model was treated with different preparations every 3 days for a total of four times. The mice model of progressive phase was developed after 14 days of BLM administration. The grouping and treatment process was the same as the injury phase of lung tissue.

Peripheral blood of different phases of PF mice was taken from eye vein plexus bleeding once every 7 days to analyze the proliferation of inflammatory cells.

After treatment, PF mice were euthanized and one lobe of the lung tissue was stored in liquid nitrogen for biochemical index detection, and other lung tissues were fixed in 4% paraformaldehyde for immunohistochemical evaluation.

### Treatment of PF Mice—Characterization of Lung Morphology Using Micro‐CT

The PF mice were anesthetized using isoflurane, and the morphology of lungs was performed using micro‐CT in vivo (PINGSENG SCIENTIFIC, Super Nova, China). The original data obtained from the scan were 3D reconstructed through Avatar provided by PINGSENG Healthcare.

### Treatment of PF Mice—Proliferation of Inflammatory Cells

During the treatment, peripheral blood was taken weekly in each group and performed for whole blood analysis (Mindray, BC‐2800vet, China).

### Treatment of PF Mice—Detection of Biochemical Indicators

The frozen lung tissues were homogenized to measure the content of biochemical indicators, including VEGFR, caspase‐3, MMP‐2, MMP‐9, CTGF, TGF‐β, and IL‐1β by ELISA assay. The operation procedures included sample pretreatment, drawing of standard curves, and sample testing were performed according to instructions of ELISA kits.

The content of SOD, GSH, MDA, iNOS, and HYP were carried out according to the specific instructions of relevant kits of Nanjing Jiancheng Biological.

### Treatment of PF Mice—Wet‐Dry Weight Ratio in Lung Tissue

The lung tissues were removed and the wet weight was recorded. Then, the lung tissues were put and dried at 80 °C for 96 h, which were recorded as dry weight. The ratio of wet weight to dry weight was calculated and the data were sorted and analyzed.

### Treatment of PF Mice—H&E Staining and MASSON Staining

The fixed lung tissue was embedded, and then the paraffin microtome was used to cut out 4 µm sections for H&E and Masson staining to evaluate the structural changes and collagen deposition of lung tissue. The Ashcroft score was obtained by the results of H&E and Masson staining.^[^ ^20^ ^]^

### Treatment of PF Mice—Mitochondrial Morphologies of Injured AECs II In Vivo

After the treatment, the mice were euthanized, and lung tissue of 5 mm^3^ was fixed with 2.5% glutaraldehyde. The mitochondria in injured AECs II was observed by TEM.

### Treatment of PF Mice—RNA Transcriptome Sequencing

The PF mice were euthanized, and the lung tissues were put into the frozen tube. RNA sequencing, bioinformatics and annotations RNA sequencing was performed by Novogene Co., Ltd. (Beijing, China) using the NovaSeq X Plus Series PE150 platform.

### Statistical Analysis

In statistical analysis, the one‐way ANOVA (Tukey test) was used in more than three groups and Student's *t*‐test was used to analyze the difference between two experimental groups. No significant difference (n.s.): *p* > 0.05, *p < 0.05 was considered to have differences, **p < 0.005 was considered to have significant differences, ***p < 0.001 was considered to have extremely significant differences. During the experiment, repeated experiments were followed, and each group contained at least three samples. All data were presented as mean ± SD. The data analysis was performed using Graphpad prism 8.0.

## Conflict of Interest

The authors declare no conflict of interest.

## Author Contributions

Q.L.L, X.C., Y.M.H, and Z.C.G contributed equally to this work. X.C. conceptualized the study and designed experiments. Q.L.L., Y.M.H., and Y.N.L. performed experiments. Q.L.L., Z.C.G., B.R.Y., and C.L. analyzed the data. X.C., Z.K.F., H.L.J., and B.G wrote the manuscript. All authors contributed to interpreting the results.

## Supporting information



Supporting Information

## Data Availability

The data that support the findings of this study are available from the corresponding author upon reasonable request.
