# Supplementary material for: Consumption of Endogenous Caspase‐3 Activates Molecular Theranostic Nanoplatform against Inflammation‐Induced Profibrotic Positive Feedback in Pulmonary Fibrosis
Source: Adv Sci (Weinh). 2024 Dec 17;12(6):2412303. doi: 10.1002/advs.202412303 (PMC11809389; doi:10.1002/advs.202412303)
Supplement: Supplementary file 1 — Supporting Information [file ADVS-12-2412303-s001.docx]

*Supporting Information*

**Consumption of Endogenous Caspase-3 Activates Molecular Theranostic Nanoplatform Against Inflammation Induced Profibrotic Positive Feedback in Pulmonary Fibrosis**

Qiu-Ling Li^1†^, Xin Chang^1,2†^, Yu-Mo Han^1†^, Zi-Chao Guo^3†^, Yi-Na Liu^1^, Bin Guo^1,2^, Chang Liu^1^, Bin-Rong Yang^1,2^, Zhong-Kai Fan^3*^, Hu-Lin Jiang^4,5,6*^, Xin Chang^1,2*^

Qiu-Ling Li, Xin Chang, Yu-Mo Han, Yi-Na Liu, Bin Guo, Chang Liu and Bin-Rong Yang

School of Pharmacy, Jinzhou Medical University, Jinzhou, Liaoning 121001, China;

Xin Chang, Bin Guo

Liaoning Provincial Key Laboratory of Marine Bioactive Substances, Jinzhou Medical University, Jinzhou, Liaoning 121001, China.

Zi-Chao Guo, Zhong-Kai Fan

The First Affiliated Hospital of Jinzhou Medical University, Jinzhou Medical University, Jinzhou, Liaoning 121001, China.

Hu-Lin Jiang

State Key Laboratory of Natural Medicines, China Pharmaceutical University, Nanjing, Jiangsu 210009, China.

Hu-Lin Jiang

Jiangsu Key Laboratory of Druggability of Biopharmaceuticals, China Pharmaceutical University, Nanjing, Jiangsu 210009, China.

Hu-Lin Jiang

NMPA Key Laboratory for Research and Evaluation of Pharmaceutical Preparations and Excipients, China Pharmaceutical University, Nanjing, Jiangsu 210009, China.

^†^These authors contributed equally to this work.

^*^Correspondence to: Xin Chang (cx0924@jzmu.edu.cn); Hu-Lin Jiang (jianghulin3@cpu.edu.cn); Zhong-Kai Fan (fanzk@jzmu.edu.cn).**I. Supporting Figures**

**
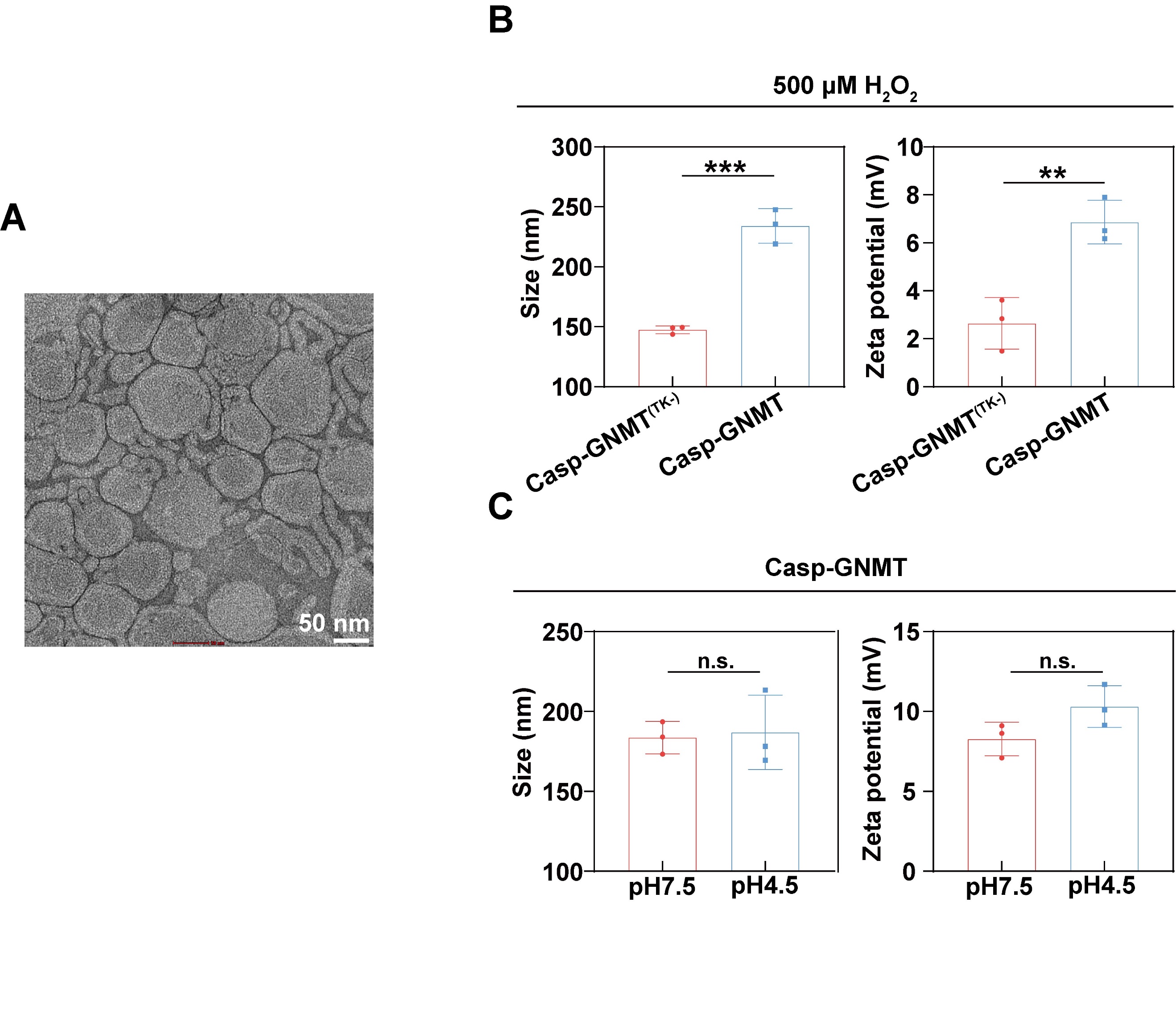
**

**Figure S1. The characterization of the nanoplatform.** A) The image of transmission electron microscope (TEM) of Casp-GNMT. B) Changes of particle sizes and zeta potential of Casp-GMT^(TK-)^ and Casp-GMT at 500 μM H_2_O_2_ (n = 3). C) Changes of particle sizes and zeta potential of Casp-GMT^(TK-)^ and Casp-GMT at pH7.5 and pH4.5 after 48 h (n = 3). The data in each panel was represented by mean ± SD by Student’s t test. No significant difference (n.s.): P > 0.05, **p < 0.005, ***p < 0.001.

**
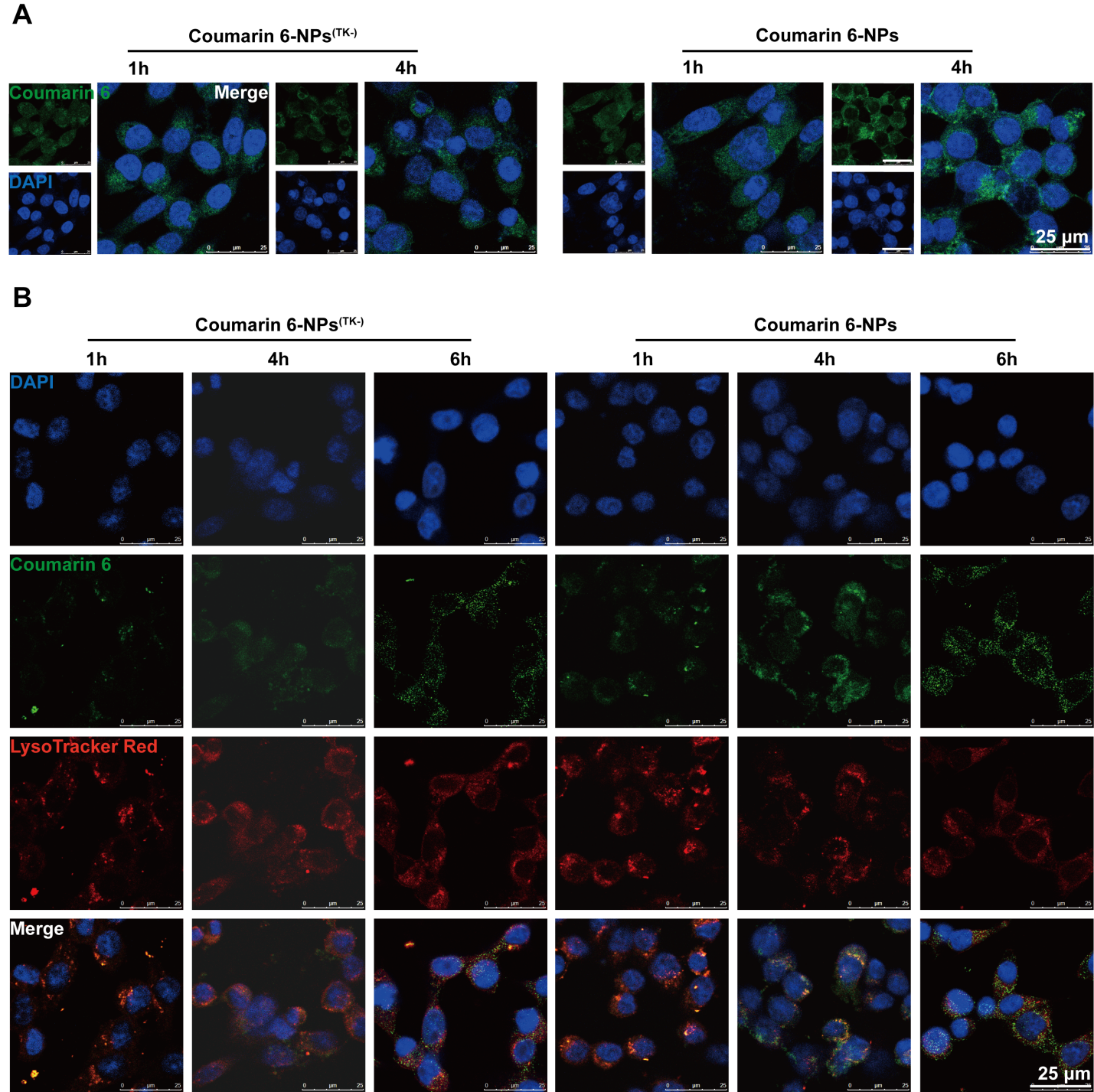
**

**Figure S2.** **Uptake efficiency and lysosome escape capacity of Coumarin 6-loaded NPs.** A) Uptake efficiency of Coumarin 6-NPs and Coumarin 6-NPs^(TK-)^ by confocal laser scanning microscope (CLSM). B) Lysosome escape capacity of Coumarin 6-NPs and Coumarin 6-NPs^(TK-)^ by CLSM.

**
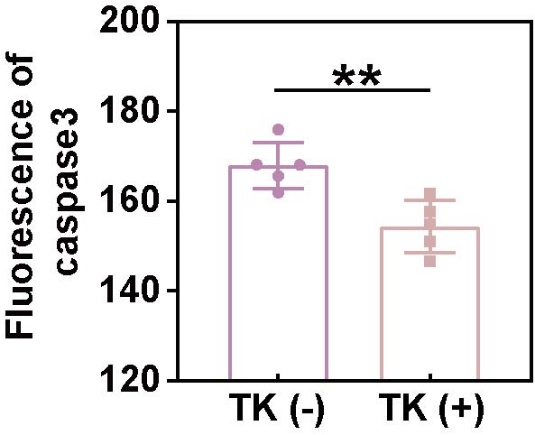
**

**Figure S3.** The expression of cysteinyl aspartate specific proteinase-3 (caspase-3) after treating with different preparations (n = 5). The data in each panel was represented by mean ± SD by Student’s t test, **p < 0.005.


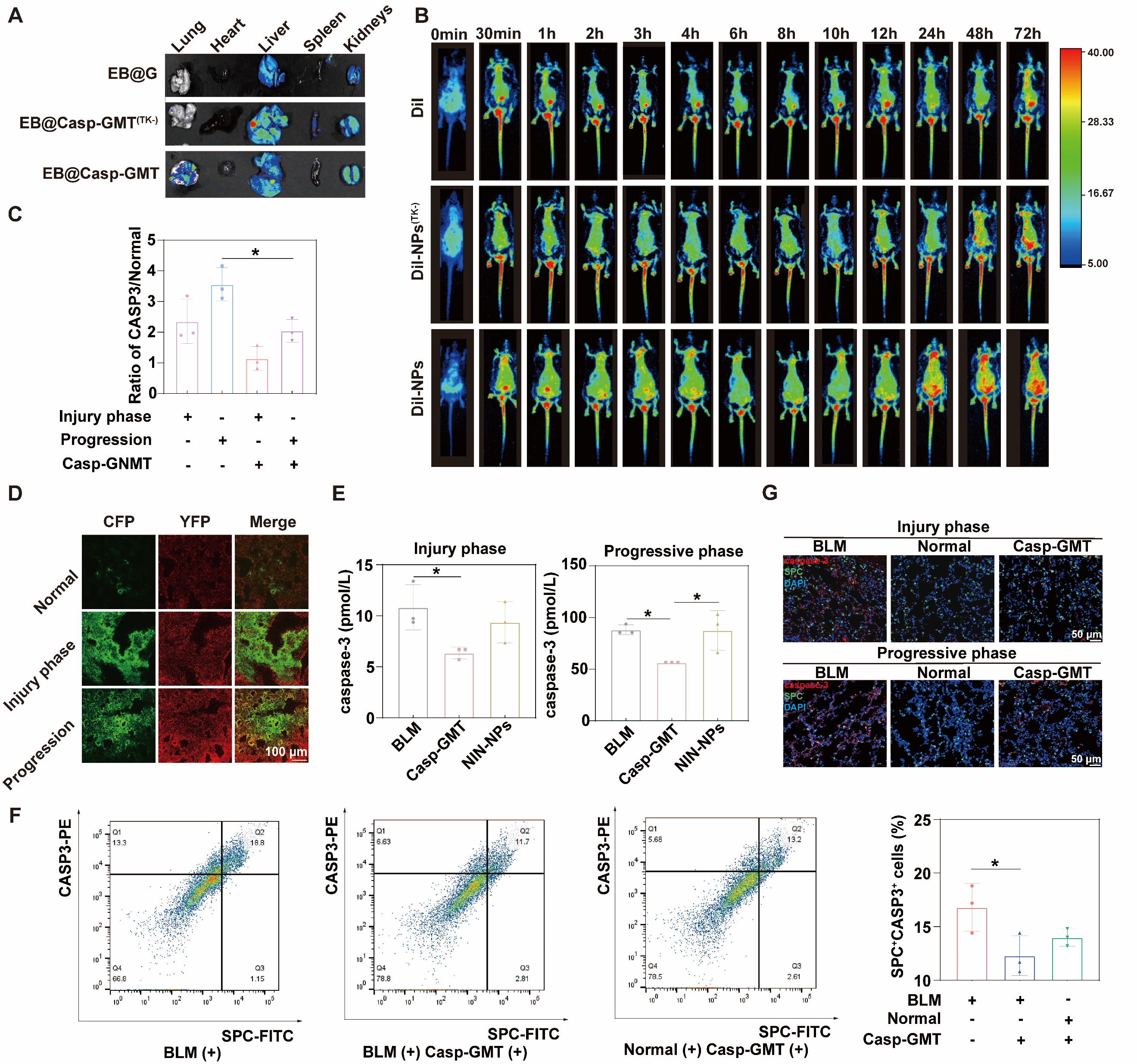


**Figure S4.** A) The *ex vivo* fluorescence intensity of lung, heart, liver, spleen and kidneys of EB@G, EB@Casp-GMT^(TK-)^ and EB@Casp-GMT at 3 h. B) Biodistribution of DiI, DiI-NPs^(TK-)^ and DiI-NPs at different time points. C) The expression level of caspase-3 before/after treatment of Casp-GNMT by ELISA assay. D) The diagnostic sensitivity of Casp-GNMT in injury and progressive phases of PF by two-photon CLSM. E) The content of caspase-3 after diagnosis in injury and progressive phases of PF. F,G) The expression level of caspase-3 in injured alveolar epithelial cells II (AECs II) by fluorescence activated cell sorting (F) and immunofluorescence staining (G) after treating with Casp-GMT. The data in each panel was represented by mean ± SD by One-Way ANOVA, *p < 0.05.


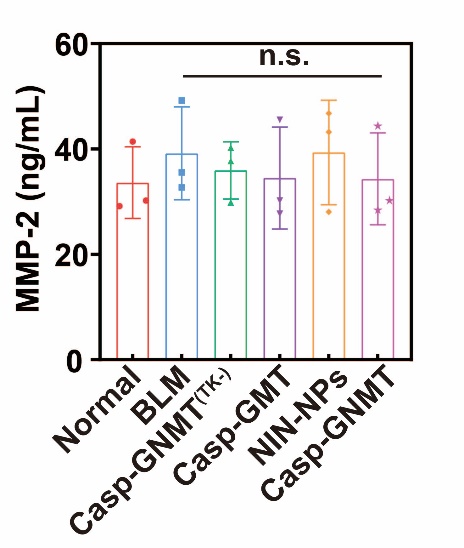


**Figure S5. Therapeutic efficacy of Casp-GNMT and other treatments in injury phase of PF.** Content of matrix metalloproteinase-2 (MMP-2) by ELISA assay (n = 3). The data was represented as mean ± SD by One-Way ANOVA. No significant difference (n.s.): P > 0.05.


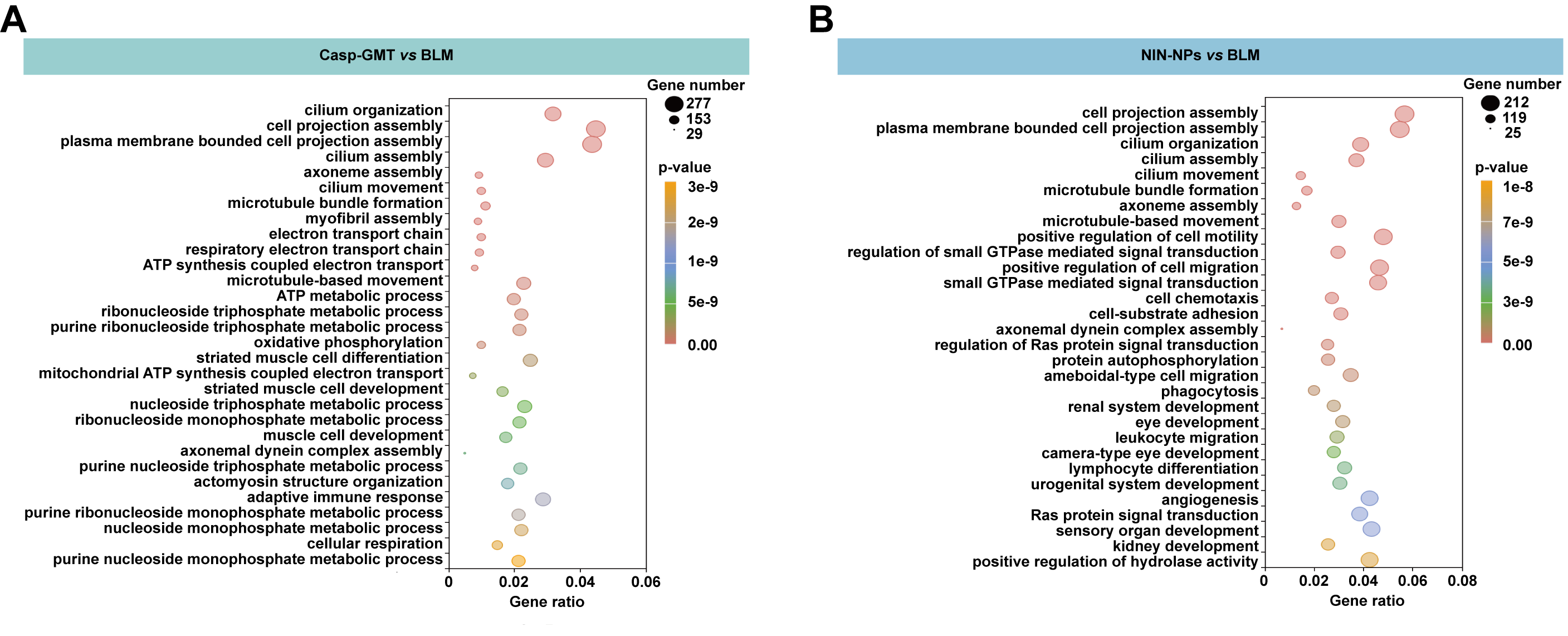
**Figure S6. Differential genes analysis of Casp-GMT *vs* BLM and NIN NPs *vs* BLM.** A) The top 30 terms in BP between Casp-GMT *vs* BLM. B) The top 30 terms in BP between NIN-NPs *vs* BLM.


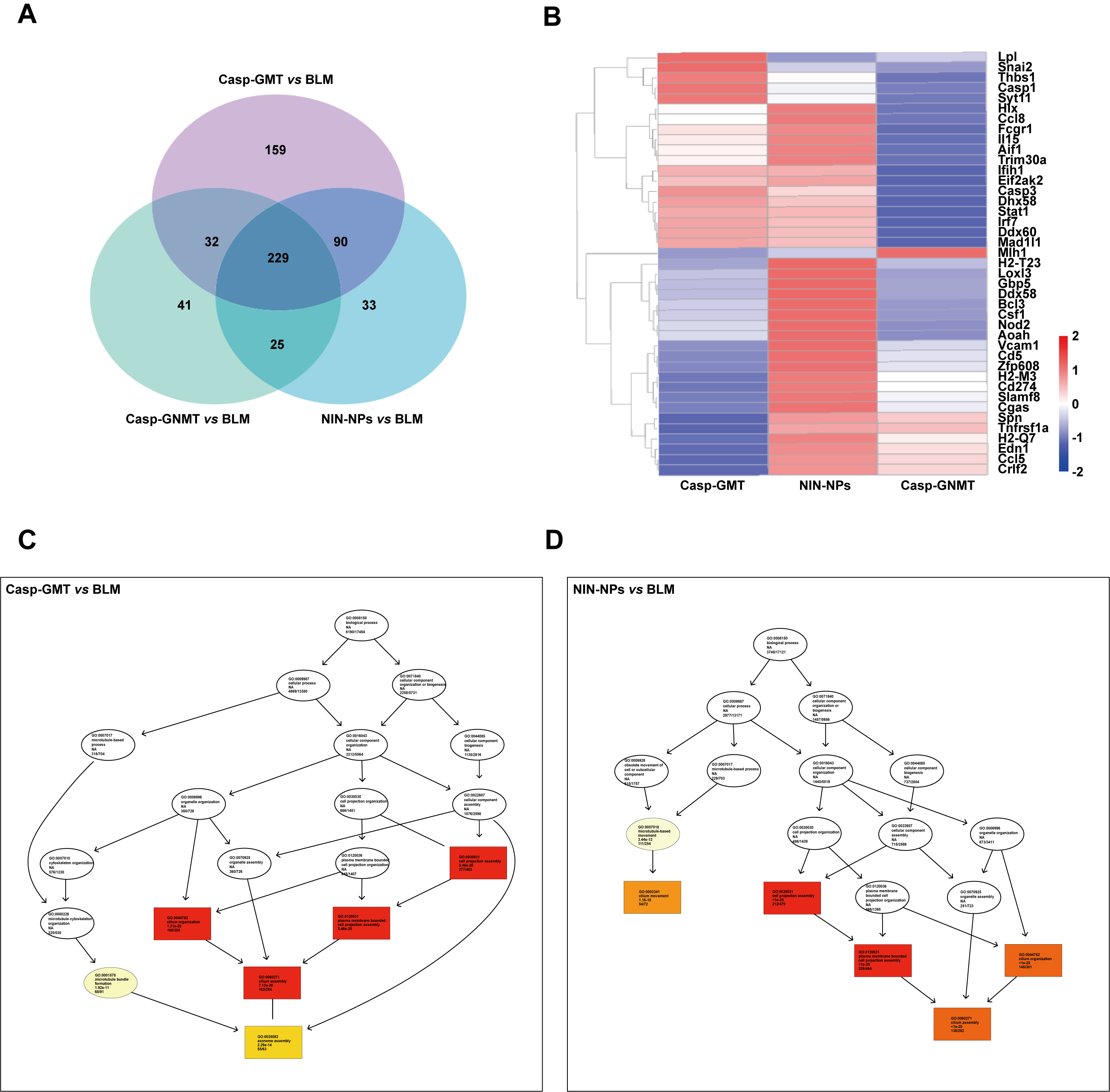
**Figure S7. Therapeutic mechanism of Casp-GNMT in precision theranostic manner.** A) Venn diagrams showed the distribution of differential genes in Casp-GNMT vs BLM, Casp-GMT vs BLM, NIN-NPs vs BLM in top 10 terms associated with inflammation based on GO analysis. B) Gene cluster analysis in top 10 terms associated with inflammation among Casp-GNMT, Casp-GMT, NIN-NPs and BLM. C,D) The mode of action of antifibrosis in Casp-GMT *vs* BLM (C) and NIN-NPs *vs* BLM (D).


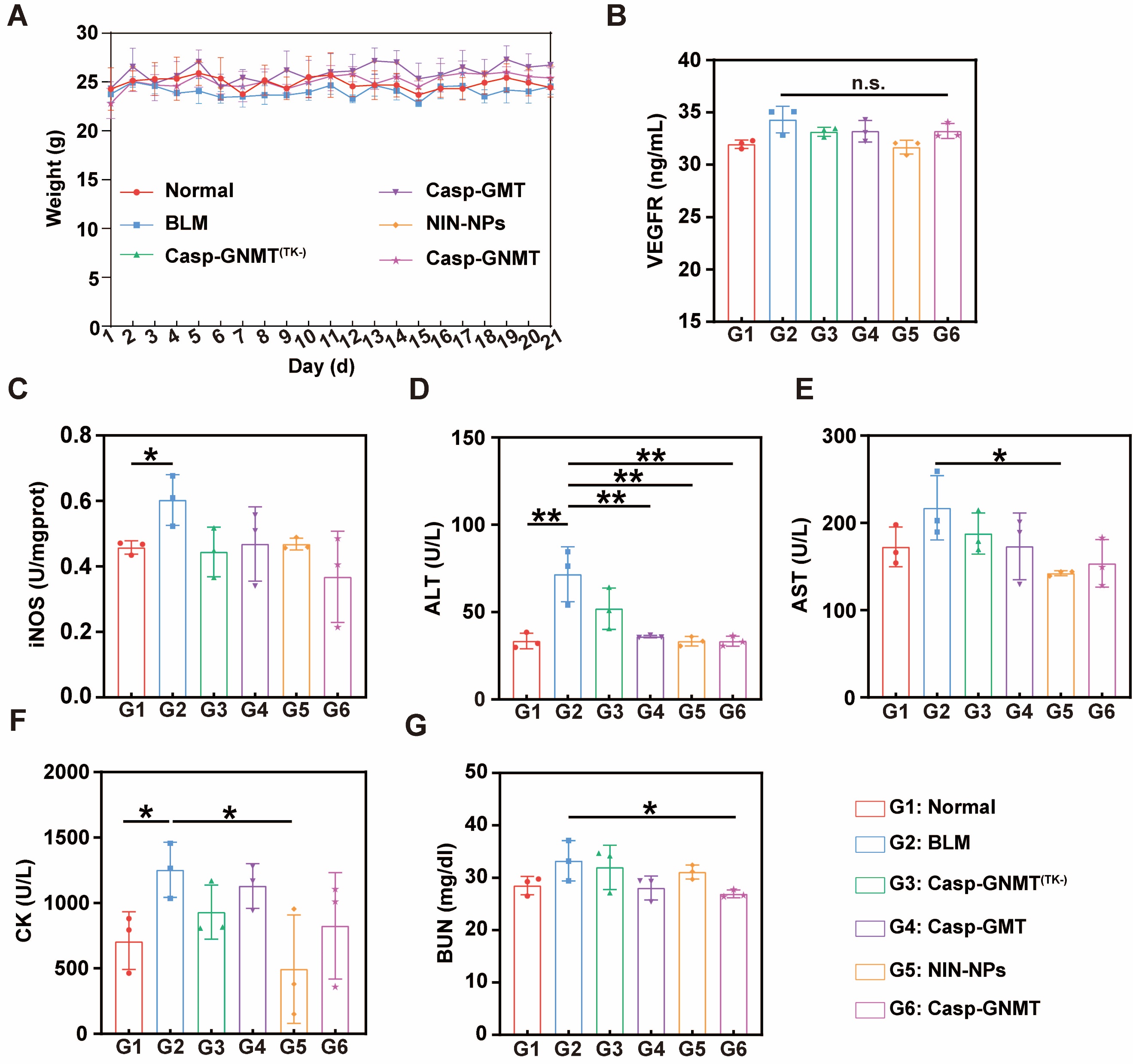


**Figure S8. Treating efficacy of different treatments in progressive phase of PF mice.** A) Changes of body weight during treatment (n = 3). B) Expression of vascular endothelial growth factor receptor (VEGFR) in different treatments by ELISA assay (n = 3). C) Content of inducible nitric oxide synthase (iNOS) by detection kit (n = 3). D-G) Biosafety of Casp-GNMT^(TK-)^, Casp-GMT, NIN-NPs, Casp-GNMT, hepatotoxicity (D and E), cardiotoxicity (F) and nephrotoxicity (G) (n = 3). The data was represented as mean ± SD by One-Way ANOVA. No significant difference (n.s.): P > 0.05, *p < 0.05, **p < 0.005.
